# Co-application of biochar and melatonin enhances rice resilience to combined lead and microplastics stress via modulating antioxidant defense, hormonal regulation, gene expression, and soil quality

**DOI:** 10.1186/s12870-026-08760-y

**Published:** 2026-04-22

**Authors:** Jie Du, Wenjie Yang, Jiayong Liu, Zhixuan Du

**Affiliations:** 1 Department of Biological and Food Engineering, Lyuliang University, Lyuliang, 033000 China; 2 School of Life Sciences, Key Laboratory of Jiangxi Province for Biological Invasion and Biosecurity, Jinggangshan University, Ji’an, 343009 China

**Keywords:** Biochar, Gene expression, Hormonal balance, Melatonin, Soil fertility, Yield

## Abstract

**Background:**

Microplastics (MPs) pollution is becoming a serious challenge and poses ecological risks. MPs are known to interact with heavy metals, such as lead (Pb), adversely impacting plant growth. Both biochar (BC) and melatonin (MT) have been extensively utilized in soil remediation efforts. However, the combined effect of BC and MT in alleviating the combined toxicity of Pb and MPs has not been investigated.

**Methodology:**

The study included various treatments: T_1_: control, T_2_: Pb (250 mg kg^− 1^) + MPs (1%), T_3_: Pb (250 mg kg^− 1^) + MPs (1%) + BC (2%), T_4_: Pb (250 mg kg^− 1^) + MPs (1%) + MT (100 µM) and T_5_: Pb (250 mg kg^− 1^) + MPs (1%) + BC (2%) + MT (100 µM).

**Results:**

Lead + MPs reduced the rice biomass yield (BY: 25.26%) and grain yield (GY: 41.51%) by increasing the hydrogen peroxide (H_2_O_2_), malondialdehyde (MDA), Pb accumulation, and decreasing the indole acetic synthesis (IAA), gibberellic acid synthesis (GA), transpiration rate (Tr), photosynthetic rate (Pn), stomatal conductance (Gs), intercellular CO_2_ (Ci), soil nitrogen, phosphorous and potassium availability. The combination of BC + MT enhanced rice BY (18.20%) and GY (39.59%) by increasing IAA (36.75%), GA (41.90%), Pn (32.35%), Tr (37.15%), gs (44.44%), and Ci (20.69%) and decreasing the production of MDA and H_2_O_2_ by increasing the antioxidants activity (41.17–52.55%). Furthermore, the combined application of BC and MT resulted in the upregulation of antioxidant genes (*OsAPx6*, *OsCAT*, *OsPOD*, and *OsSOD*), melatonin synthesis gene (OsASMT1), and proline biosynthesis gene (OsP5CS), while concurrently downregulating genes associated with lead uptake (*OsHMA9* and *OsNRAMP5*). The BC + MT treatment also improved soil total nitrogen (TN) by 28.19%, available phosphorus (AP) by 26.73%, and available potassium (AK) by 18.81%, alongside an increase in soil pH. Additionally, it reduced soil lead availability by 37.18%, collectively contributing to enhanced rice biomass and grain yield.

**Conclusion:**

Thus, BC + MT can alleviate adversities of Pb + MPs by improving soil fertility, plant functioning, hormonal balance, and gene expression. These findings offer insights to develop measures to enhance the crop in multi-polluted soils.

**Supplementary Information:**

The online version contains supplementary material available at 10.1186/s12870-026-08760-y.

## Introduction

Plastics have extensive use in the packaging, electronics, and textile industries due to their lightweight nature and resistance to corrosion and water [1]. The prevalence of plastics has markedly increased in society, with production reaching 413.8 million tons in 2023 [2], leading to their accumulation in soils in the form of microplastics (MPs). Key pathways for the introduction of MPs into agricultural environments include irrigation water, sewage sludge, sewage irrigation, sludge-based fertilizers, mulching, coated fertilizers, and atmospheric deposition [3, 4]. Microplastics exhibit a diverse range of sizes, shapes, persistence, chemical properties, and ecological impacts [4]. The effects of MPs on aquatic ecosystems have been extensively researched, however, their impact on terrestrial ecosystems remains comparatively less studied [5]. Microplastics exert phytotoxic effects by enhancing the availability of toxic metals and generating hazardous compounds [6]. Additionally, they influence soil structure, soil organic carbon (SOC) concentration, soil pH, and reduce nutrient availability [7]. Microplastic decreases plant growth by decreasing seed germination, photosynthetic efficiency, and inhibiting root growth [8, 9]. They also restrict the nutrient and water absorption and negatively affect the plant physiological and metabolic functioning [10, 11]. Additionally, they also damage cell structure and negatively affects the soil microbial and enzyme activity, leading to poor growth [12].

Adding to this longstanding challenge, heavy metals pollution is also a serious threat to crop productivity. Lead (Pb) is a toxic metal and its accretion in soils increasing due to industrial processes, transportation, Pb-petroleum, and agricultural activities, thereby posing significant risks to plants and humans [13]. It disrupts the soil physiochemical properties, hinders root growth, and photosynthetic efficiency, consequently reducing the growth [14]. Soil contamination with MPs and Pb represents a crucial challenge to soil-plant ecosystems [15, 16]. Recent studies reported that MPs synergistically enhanced the Pb accumulation in plants and facilitated its entry into food chains, thereby posing a health risk in humans [14, 17]. The excessive intake of Pb causes heart diseases, nervous system damage, pulmonary, and hepatic disorders [18]. In plants Pb cause cellular damages, decreases photosynthetic efficiency, nutrient uptake and biomass yield [19]. The co-exposure cause more severe damage than individual stresses by increasing membrane damage, oxidative stress, and compromising the antioxidant activities [20, 21]. These studies indicate that co-exposure of Pb and MPs can pose more toxic impacts to plants; therefore, it is essential to put forth measures to counteract the combined Pb and MPs toxicity in plants. 

Biochar (BC) is a carbonaceous product produced by pyrolysis, and it has been acknowledged as an important amendment to mitigate climate change and hazardous impacts of toxic pollutants [22]. Biochar improves soil health via increasing microbial and enzyme activity, nutrient availability, thus promoting stress resilience [23]. It also improves antioxidant activity, metabolic functioning, chlorophyll synthesis, osmolytes production, glucose metabolism, and plant transcriptomic responses, and improves the plant growth in Pb and MPs-polluted soils [4, 24, 25]. The efficiency of BC to alleviate Pb and MPs toxicity is largely depends on BC and soil properties, intensity, and duration of stress. Recently, BC has been used in combination with other amendments to mitigate the adversities of abiotic stresses. The combination of BC with phyto-hormones and signaling molecules has emerged as an important strategy to mitigate adversities of abiotic stress [26]. Melatonin (MT) holds great promise for enhancing growth and stress resilience [27, 28]. Melatonin favors chlorophyll synthesis, maintains water relationships, osmolytes synthesis, and hormonal balance, contributing to better growth in stress conditions [29, 30]. Melatonin mitigates heavy metals and MPs’ toxicity by decreasing their uptake and accumulation, scavenging ROS, and maintaining better redox homeostasis [28, 31]. Recently, co-applied BC and MT showed better results in mitigating Pb and As toxicity in rice via increasing proline synthesis, antioxidant activities, gene expression, and soil nutrients availability [25, 28].

Rice is serving as an important staple food crop globally; nevertheless, MPs and toxic metals are negatively affecting rice productivity, posing huge challenges to sustainable and safe rice production [32]. Microplastics enter the rice fields through irrigation water, plastics, and fertilizers, and the presence of MPs in rice plants and paddy is reported in recent studies [32, 33]. In the literature, the roles of BC and MT in mitigating single heavy metal contamination, such as cadmium and Pb, are well documented; however, their effectiveness in the presence of MPs remains unexplored. Microplastics serve as vectors for toxic metals and alter soil properties, thereby influencing the availability and toxicity of Pb. This complexity may affect the efficacy of BC and MT in addressing co-contamination. Consequently, this is the first study to investigate the efficiency of BC and MT in enhancing plant growth and reducing Pb accumulation under dual contamination by MPs and Pb, thus addressing a critical knowledge gap in multi-contaminant soil remediation. The study aims to extend the existing foundation by introducing 1% MPs as an additional variable to better simulate real-world co-contamination scenarios. We hypothesized that co-applied BC and MT synergistically enhance rice growth, yield, and tolerance against combined Pb and MPs toxicity. The study aimed: (1) to determine impacts of BC + MT on rice yield and plant functioning (2), to assess how BC and MT affect hormonal regulation, endogenous MT synthesis, nutrient balance and Pb accumulation in rice plants (3) to explore the effects BC + MT on gene expression levels and soil fertility to counteract combined Pb and MPs toxicity.

## Materials and methods

### Experiment site and materials preparation

The soil was taken from Jinggangshan University. The soil was firstly sieved (2 mm) to remove the debris, and later pots were filled. The soil was classified as silt loam (5.55: pH), furthermore, the soil had a TN concentration of 1.81 g kg^− 1^ and AP and AK concentrations of 29.12 mg kg^− 1^ and 114.29 mg kg^− 1^, respectively. Furthermore, the soil had a cation exchange capacity (CEC) of 7.34 cmol kg^− 1^ and organic carbon contents of 10.62 g kg^− 1^, respectively. The preparation of BC involved pyrolyzing collected rice straw at 500 °C under controlled atmospheric conditions. Biochar was sieved and subjected to measuring different properties before using it in the experiment. Biochar had a pH of 9.63, and further details are given in results section.

### Experiment setup

A completely randomized design was utilized in this study consisting of three replicates for every treatment (*n* = 3). The study was performed from August to October, 2025. The experiment contained the following treatments: The study included various treatments: T_1_: control, T_2_: Pb (250 mg kg^− 1^) + MPs (1%), T_3_: Pb (250 mg kg^− 1^) + MPs (1%) + BC (2%), T_4_: Pb (250 mg kg^− 1^) + MPs (1%) + MT (100 µM) and T_5_: Pb (250 mg kg^− 1^) + MPs (1%) + BC (2%) + MT (100 µM). The polyvinyl chloride MPs were used in the study, which have a size of 10 μm and a density of 1.40 g cm^− 3^. The rate of MPs was 1% because 1% MPs is mostly used in earlier studies [34]. The rate of BC was 2% and it was selected from previous studies showing that this rate mitigates MPs’ toxicity and it is also consistent with the practical application level of BC [4, 35]. The rates of Pb was also selected from earlier studies of Yang et al. [26] and it was achieved with (Pb (NO_3_)_2_). The soil allowed to stabilize by placing it in dark conditions for two months, while maintaining a field capacity of 70%. Subsequently, MPs were added to the soil, stirred thoroughly, and left to equilibrate for a period of two weeks. Biochar thoroughly mixed with soil and allowed to equilibrate for 2 weeks, and after this pots were filled with soil. The rice seeds were purchased from the local market, and the nursery was grown and 25 days old seedlings were transplanted in every pot. All pots were maintained under regular irrigation with a consistent water level of 2–3 cm. At 20 days after transplanting, MT was applied was foliar spray for three consecutive days. Briefly MT was dissolved in minimum volume of ethanol and then it was diluted by using the distilled water. Then it was sprayed by adding the surfactant named as Tween-20. The rate of MT was selected from previous studies reporting that foliar applied MT (100 µM) showed effective results in mitigating heavy metals toxicity [28, 36–38].

### Plant samples analysis

The five rice plants were harvested at maturity and separated into roots and shoots, to measure their lengths as well biomass with standard procedures. To determine dry biomass, samples were oven-dried (65 °C) until a constant weight. The same plants were utilized for measuring, plant height, panicle length, grains/panicle, grain weight, biomass, and grain yield and harvest index with standard procedures.

### Measurement of leaf photosynthetic traits and oxidative stress markers

The concentration of chlorophyll (Chl) in rice leaves was determined with the methods of Arnon et al. [39]. For this, 0.2 g leaf samples were extracted with 95% ethanol (10 mL). The mixture was placed in the dark overnight, and after this, it was centrifuged, and an extract was obtained, which was then used to measure the absorbance at 665, 649, and 470 nm for determining chlorophyll (Chl) a, Chl-b, and carotenoid contents. Diverse leaf traits including Pn, gs, Tr and Ci were estimated with Li-6800 fluorimeter (Lincoln, NE, USA). The sampling was done from fully expanded leaves during 9–11 am. The concentration of MDA produced in the rice leaves was determined with the methods of Hodges et al. [40]. Briefly, leaves ground in 2.5 mL of thiobarbituric acid (TBA: 0.5%) using the pestle and mortar. Then, the mixture was obtained and added to 10% trichloroacetic acid (TCA) and subjected to incubation (100 °C) for half an hour. After cooling, samples subjected to centrifugation and reading recorded at 532, and 600 to measure MDA contents. For H_2_O_2_ measurement; briefly, 0.2 g of fresh leaves was ground in 1 mL TCA solution (0.1%) and centrifuged (12,000 × g) for 15 min and reading measured at 390 nM [41]. Electrolyte leakage from leaves was measured by the methods of Valentovic et al. [42] after measuring electrical conductivity twice. Leaf relative water contents (RWC) were determined based on fresh, turgid, and dry weights by following the methods of Chattha et al. [43].

### Measurement of potential osmolytes, and hormones

Fresh rice leaves (0.2 g) were ground using 50 mM PPB and then subjected to centrifugation (10,000 × g) for 15 min at 4 °C. Later this solution was mixed with 2 mL Bradford and reading measured at 595 nm [44]. Leaf proline concentration was measured by using the acid ninhydrin following the standard method of Bates et al. [45]. For endogenous MT; leaves were homogenized and added to chloroform (5 mL) and centrifuged at 10,000 rpm for 10 min. Later samples were placed in room conditions, and after evaporation of chloroform, the MT content was measured using HPLC [46]. To measure the concentration of different hormones, rice leaves were collected and stored at − 80 °C. The concentration of different hormones such as IAA, GA, and ABA was determined with a kit provided by Beijing Boxbio Science & Technology Co., Ltd, following the instructions of Koshita et al. [47].

### Antioxidants activity and gene expression analysis

Leaf superoxide dismutase (SOD) activity was measured by the methods of Zhang et al. [48]. Briefly, the reaction mixture comprising of 1.75 mL PPB, 0.3 mL methionine (130 mM), 750 µM NBT, 0.05 mL extract, and 20 µM riboflavin was prepared, and the absorbance recorded at 560 nm. The activity of peroxidase (POD) in rice leaves was measured with the methods of Xia et al. [49] after reading absorbance at 470 nm. To measure the catalase (CAT) activity, 1.95 mL phosphate buffer, 1 mL H_2_O_2_ (0.1 M), and 0.05 mL extract mixed and reading done at 240 nm [50]. For ascorbate peroxidase (APX), 100 mL extract and buffer and 1 mL H_2_O_2_ (0.06 mM) were mixed and absorbance read at 290 nm. RNA from collected rice leaves was extracted using the extraction kit supplied by Takara, China. The extracted RNA (2.5 µL) was reverse transcribed into cDNA with the aid of the RT SuperMix kit. Then SuperReal PreMix Plus (SYBR Green) was used to perform the qRT-PCR, and the expression levels of target genes were analyzed via the Livak and Schmittgen method [51].

### Measurement of plant nutrient and lead accumulation and soil properties

The samples were dried, milled and digested by using HClO_4_ and HNO_3_ in a 1:2 ratio, and after digestion, they were diluted by adding water. Plant lead, calcium, and magnesium contents were measured by an Atomic Absorption Spectrophotometer (AAS), while N concentration was measured with the Kjeldahl method [52], and phosphorus and potassium concentrations were measured with a spectrophotometer and flame photometer methods. The pH of collected soil samples was assessed with a pH meter in a 1:5 soil–water suspension. Soil total nitrogen was measured according to the Kjeldahl procedure [52], while AP and AK were measured with a spectrophotometer and flame photometer techniques [53, 54]. For measuring soil Pb concentration, samples were digested using a mixture of acids (HClO_4_ and HNO_3_) on a hotplate (160 °C). Then, they were diluted and later Pb concentration was estimated with AAS.

### Data analysis

The data on collected traits was analyzed with one-way ANOVA with Statistix 8.1. The honestly significant difference test was applied to compare treatment means, adopting a 5% significance level. The graphs used in the paper were prepared with Sigma-plot 10 and R-studio, and the relationship between different traits was analyzed with R studio.

## Results

### Characterization of biochar and micro-plastics

**Fig. 1:**
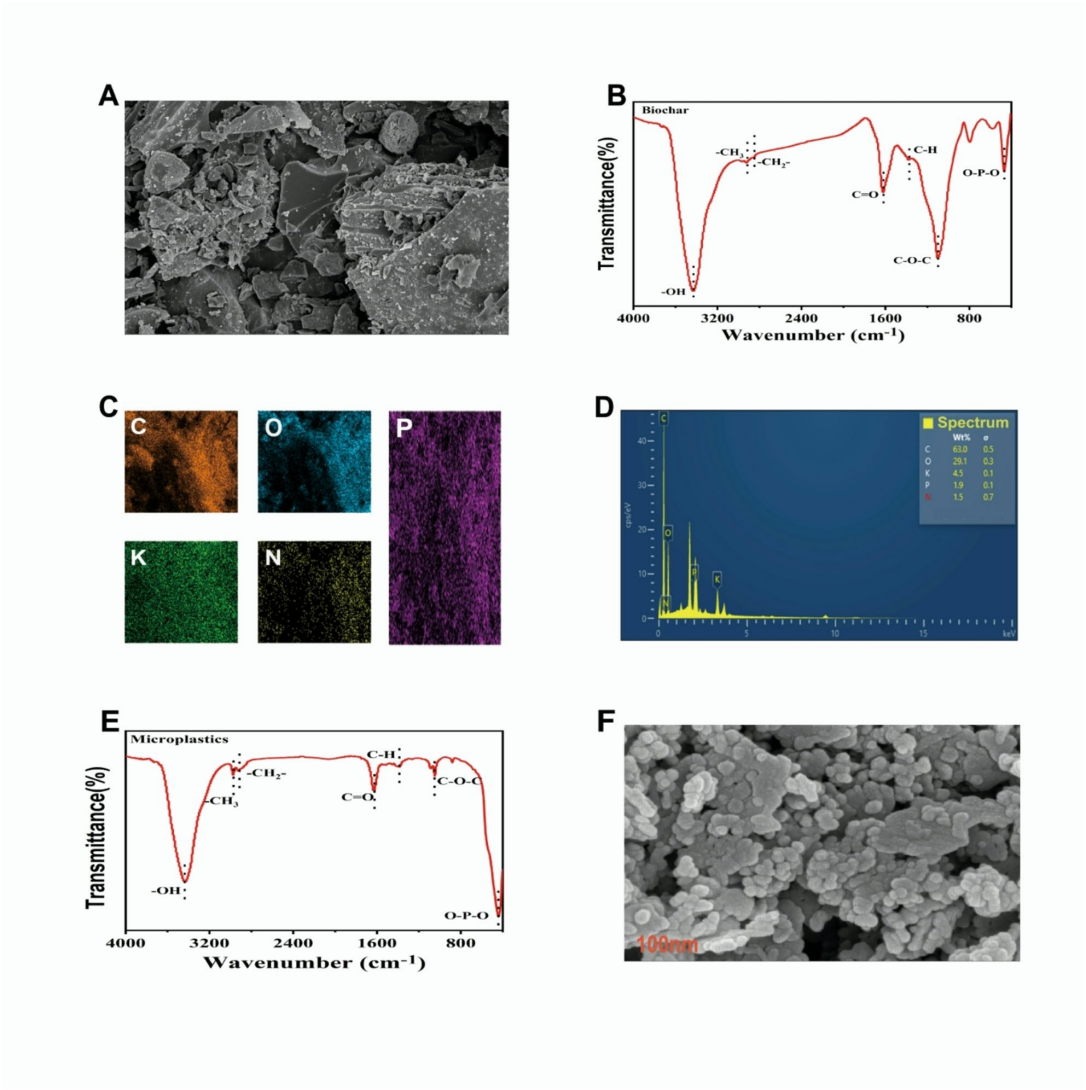
SEM (**A**), FTIR (**B**), EDS (**C** and **D**) analysis of biochar and FTIR (**E**) and SEM (**F**) analysis of microplastics used in the study

The SEM analysis revealed that BC had a flatter, rough morphology with a porous structure, showing its potential to adsorb pollutants. The FTIR analysis depicted the peak values at 3432.37, 1621.38, 1101.32, 795.09, and 467.87 cm^− 1^. The values observed at 3432.37, showing the presence of O-H groups, while values noticed at 1101.32, 795.09, and 467.87 cm^-1^ showed the presence of C = C, C-O, and C-H groups in BC. Further, EDS analysis showed that BC had carbon 63%, nitrogen 1.50%, oxygen 29.1%, phosphorus, 1.90% and potassium 4.50% with surface area of 55.16 m²/g. The results of SEM analysis showed that MPs have irregular surface structures associated with sharp edges (Fig. 1). The FTIR analysis of MPs showed the peaks at 3435.88, 2973.76, 1630.17, 1048.91, and 439.6, respectively (Fig. 1). The peaks observed at 3435.88 and 2973.76 show O-H and C-H stretching, and the peak observed at 1630.17, 1048.91, and 439.65, respectively, show the C = C stretching, C-O stretching, and C-H bending (Fig. 1).

### Morphological and growth traits

The above and below-ground traits showed a significantly (*p* ≤ 0.05) different response to Pb and MPs’ toxicity (Table 1). Compared to control, Pb and MPs considerably decreased the RL, RFW, RDW, PH, PL, GPP, 1000-GW, BY, and GY (Table 1). Biochar and MT mitigated Pb and MPs-induced toxicity and enhanced the rice below and above-ground traits (Table 1). Notably, the combined use of BC + MT enhanced below-ground traits, including RL, RFW, and RDW by 53.59%, 61.64% and 48.09% respectively, under Pb and MPs contaminated soil (Table 1). Likewise, combination of BC and MT caused a substantial increase of 25.51%, 25.67%, 27.96%, 30.58%, 18.20%, and 39.59% in PH, PL, GPP, 100-GW, BY, and GY of rice grown in Pb and MPs-polluted soil (Table 1).

**Table 1:** The effects of biochar and foliar applied melatonin on growth and morphological characteristics of rice grown in combined lead and micro-plastics contaminated soil

Treatments	RL (cm)	RFW (g)	RDW (g)	PH (cm)	PL (cm)	GPP	TGW (g)	BY (g/pot)	GY (g/pot)	HI (%)
T1	53.29a	9.67a	2.25a	105a	21.54a	124a	25.23a	178.25a	23.97a	20.97a
T2	32.17d	5.11e	1.31c	73c	14.62d	85d	15.18d	133.23d	14.02c	9.66d
T3	43.33b	7.30c	1.76b	95a	18.04bc	109bc	19.17c	152.02bc	19.12b	14.65bc
T4	38.29c	6.36d	1.66b	85b	16.98c	103c	17.11 cd	142.18 cd	17.79b	12.37 cd
T5	49.41a	8.26b	1.94ab	98a	19.67ab	118ab	21.87b	162.89b	23.21a	17.22ab

The data is mean of three replicates (*n* = 3) and same letters showing non-significant difference as determined by HSD test (0.05 probability level)

*RL* Root length, *RFW* Root fresh weight, *RDW* Root dry weight, *PH* Plant height, *PL* Panicle length, *GPP* Grains per panicle, *TGW* 1000-grain weight, *BY* Biological yield, *GY* Grain yield, *HI* Harvest index

T_1_: control, T_2_: Pb (250 mg kg^− 1^) + MPs (1%), T_3_: Pb (250 mg kg^− 1^) + MPs (1%) + BC (2%), T_4_: Pb (250 mg kg^− 1^) + MPs (1%) + MT (100 µM) and T_5_: Pb (250 mg kg^− 1^) + MPs (1%) + BC (2%) + MT (100 µM)

### Chlorophyll pigmentation and gas exchange traits

**Fig. 2:**
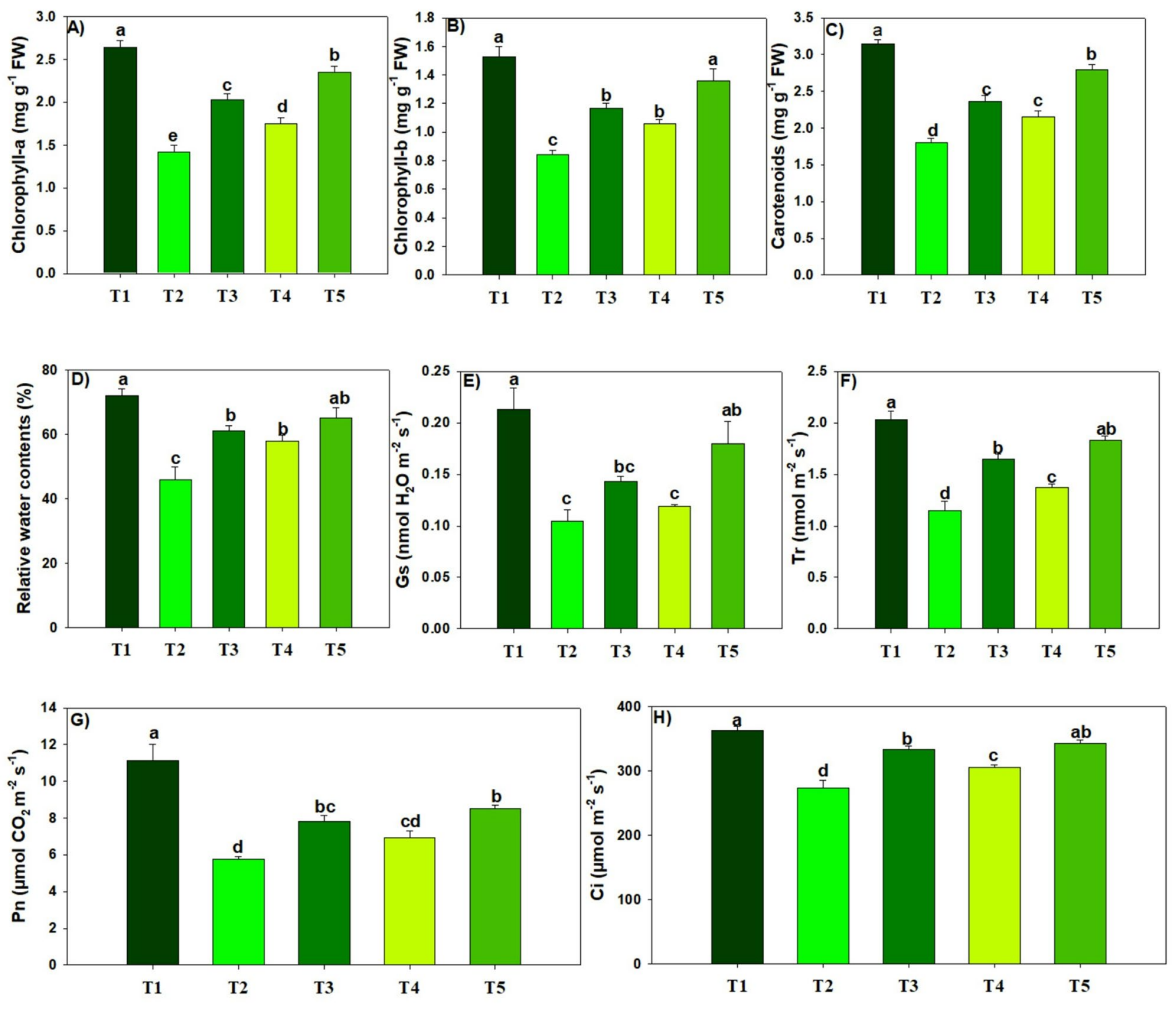
The effects of biochar and foliar applied melatonin on photosynthetic pigments (**a**-**c**), leaf water contents (**d**) and leaf gas exchange traits (**e**-**h**) of rice grown in combined lead and micro-plastics contaminated soil. The data is mean of three replicates (*n* = 3) and same letters showing non-significant difference as determined by HSD test (0.05 probability level). T_1_: control, T_2_: Pb (250 mg kg^− 1^) + MPs (1%), T_3_: Pb (250 mg kg^− 1^) + MPs (1%) + BC (2%), T_4_: Pb (250 mg kg^− 1^) + MPs (1%) + MT (100 µM) and T_5_: Pb (250 mg kg^− 1^) + MPs (1%) + BC (2%) + MT (100 µM)

The effect of different treatments was found to be significant (*p* ≤ 0.05) on Chl pigments and gas exchange traits. Overall, Pb and MPs significantly (*p* ≤ 0.05) reduced Chl concentration (Fig. 2). Biochar and MT alleviated the Pb and MPs toxicity and significantly (*p* ≤ 0.05) enhanced the chlorophyll pigments (Fig. 2). In particular, the largest increase in Chl-a (39.57%), Chl-b (38.23%), and carotenoids (35.71%) was seen with combined BC + MT treatment under Pb and MPs stress as compared to the plant receiving no BC and MT application. Of all the treatment combinations of BC and MT had the most promising effects in increasing the gas exchange traits under Pb and MPs toxicity. We found that plants receiving combined BC and MT application showed a substantial (*p* ≤ 0.05) increase in Pn (32.35%), Tr (37.15%), gs (44.44%), and Ci (20.69%) under Pb and MPs stress (Table 1). We also found that Pb and MPs significantly (*p* ≤ 0.05) decreased RWC contents (36.11%). Notably, combination of BC + MT, maintained a better RWC under Pb and MPs polluted soil than their individual application (Fig. 2).

### Stress markers and antioxidants defense

**Fig. 3:**
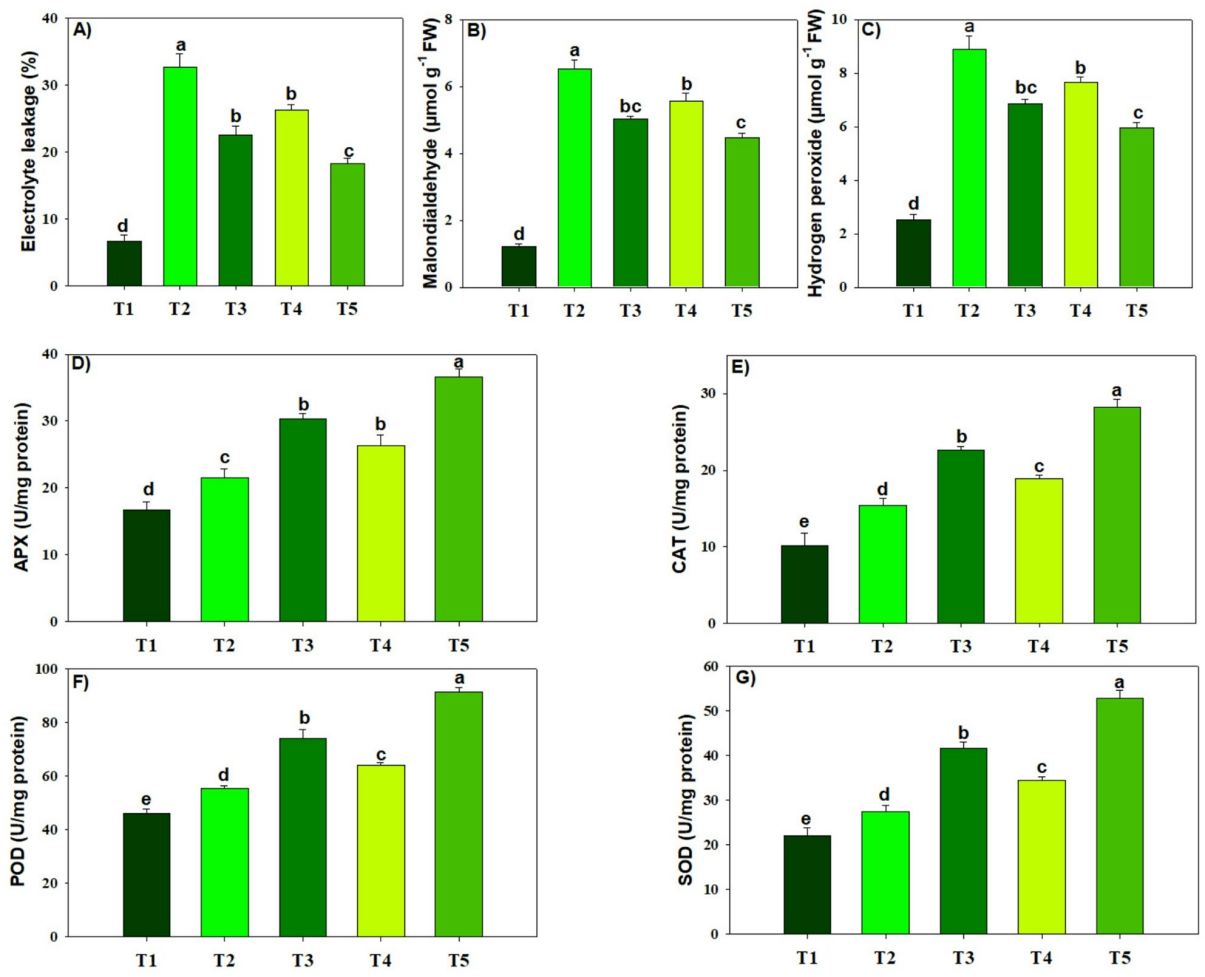
The effects of biochar and foliar applied melatonin on oxidative markers (**a**-**c**), and antioxidants activities (**d**-**g**) of rice grown in combined lead and micro-plastics contaminated soil. The data is mean of three replicates (*n* = 3) and same letters showing non-significant difference as determined by HSD test (0.05 probability level). APX: ascorbate peroxidase, CAT: catalase, POD: peroxidase, SOD: superoxide dismutase. T_1_: control, T_2_: Pb (250 mg kg^− 1^) + MPs (1%), T_3_: Pb (250 mg kg^− 1^) + MPs (1%) + BC (2%), T_4_: Pb (250 mg kg^− 1^) + MPs (1%) + MT (100 µM) and T_5_: Pb (250 mg kg^− 1^) + MPs (1%) + BC (2%) + MT (100 µM)

Lead and MPs enhanced the production of oxidative markers (Fig. 3). All amendments, such as BC and MT, decreased (*p* ≤ 0.05) the oxidative markers in response to Pb and MPs toxicity. Notably, BC + MT showed the greatest reduction of 43.86%, 31.44% and 33.18% in EL, MDA, and H_2_O_2_ in response to Pb and MPs stress (Fig. 3). The antioxidant activity was increased (*p* ≤ 0.05) in response to Pb and MPs, and it was further increased following BC and MT application. We found that the maximum improvement in APX (41.17%), CAT (45.62%), POD (52.55%), and SOD (47.85%) activity of rice plants grown in Pb and MPs soil receiving BC + MT (Fig. 3).

### Osmolytes and hormones synthesis

**Fig. 4:**
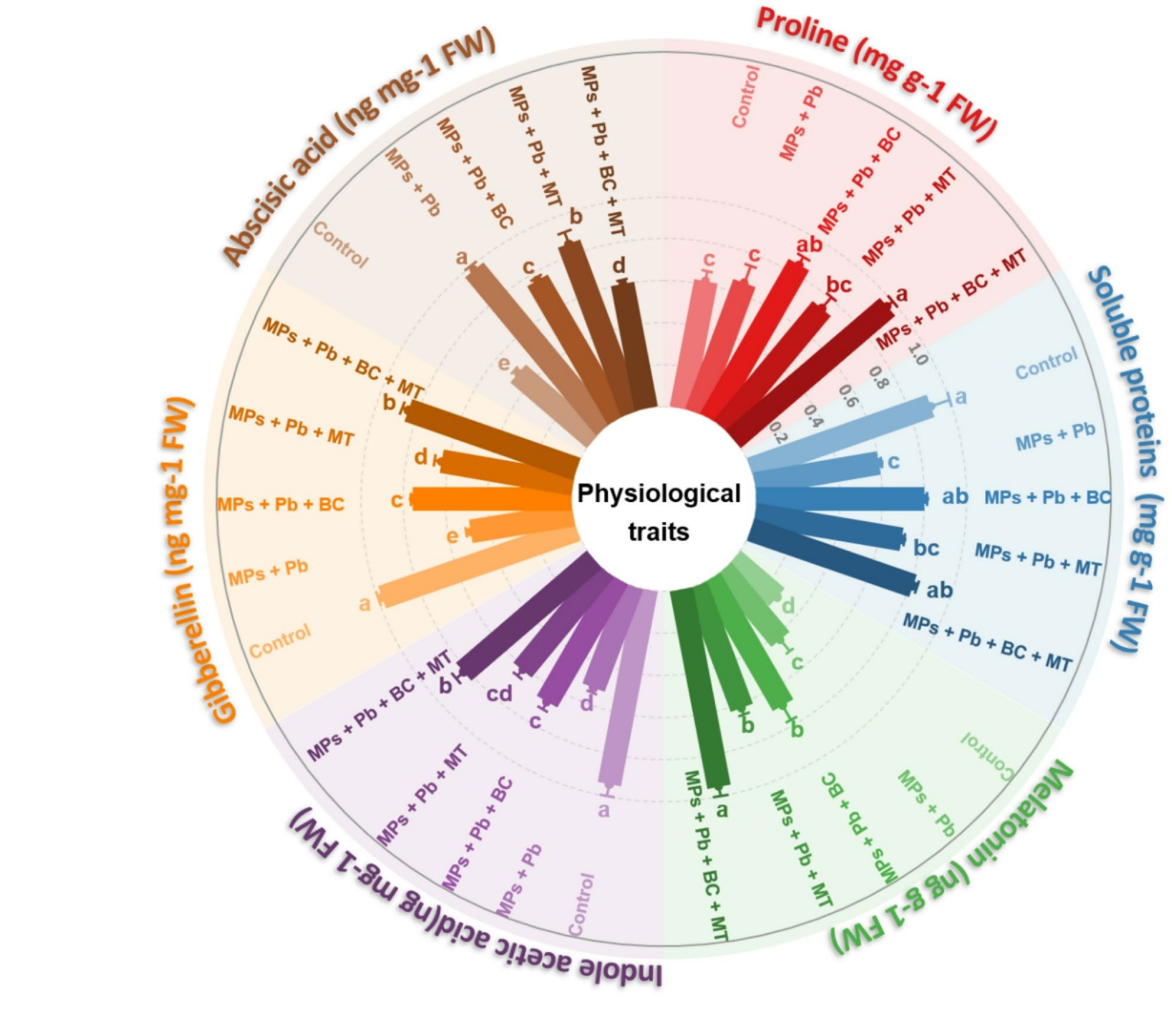
The effects of biochar and foliar applied melatonin on osmolytes and hormones of rice grown in combined lead and micro-plastics contaminated soil. The data is mean of three replicates (*n* = 3) and same letters showing non-significant difference as determined by HSD test (0.05 probability level). FW: fresh weight

Osmolytes and hormones showed a differential response to MPs and applied BC and MT (Fig. 4). In particular, synthesis of proline was increased (6.12%) under Pb and MPs, while SP synthesis was decreased (33.88%) under Pb and MPs (Table 1). The combination of BC + MT markedly increased proline and SP synthesis in response to Pb and MPs (Fig. 4). The synthesis of MT and ABA increased (*p* ≤ 0.05) under stress Pb and MPs, while BC and MT decreased the ABA synthesis, while they enhanced the synthesis of MT (Fig. 4). We noted that under Pb and MPs stress; BC + MT increased MT synthesis by 54.93% and decreased ABA synthesis by 38.16% (Fig. 4). The maximum increase of 36.75% and 41.90% in IAA and GA under Pb and MPs was observed with co-applied BC + MT (Fig. 4).

### Expression of genes associated with antioxidants and melatonin

**Fig. 5:**
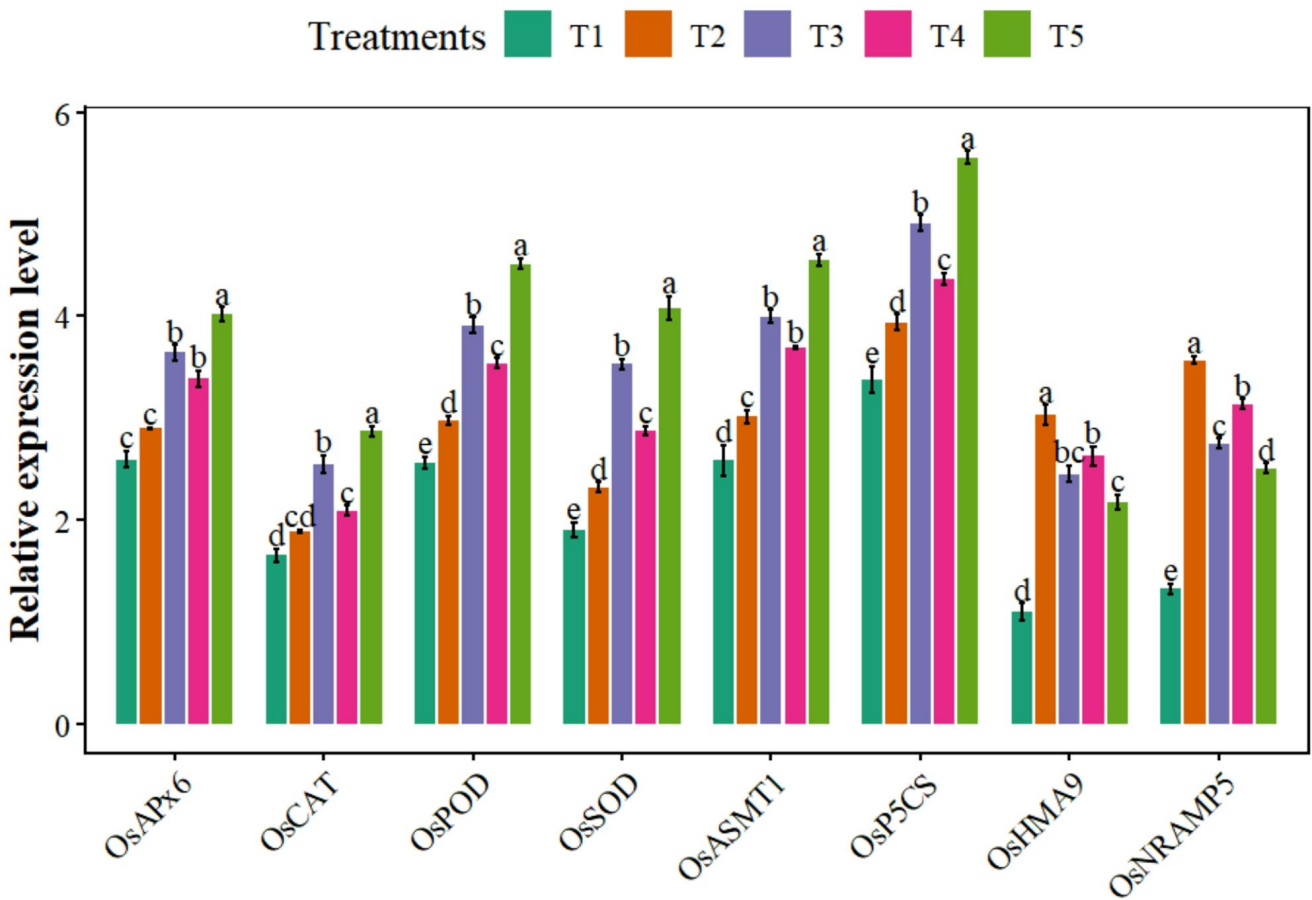
The effects of biochar and foliar applied melatonin on antioxidants, melatonin, proline and lead uptake genes expression of rice grown in combined lead and micro-plastics contaminated soil. The data is mean of three replicates (*n* = 3) and same letters showing non-significant difference as determined by HSD test (0.05 probability level). T_1_: control, T_2_: Pb (250 mg kg^− 1^) + MPs (1%), T_3_: Pb (250 mg kg^− 1^) + MPs (1%) + BC (2%), T_4_: Pb (250 mg kg^− 1^) + MPs (1%) + MT (100 µM) and T_5_: Pb (250 mg kg^− 1^) + MPs (1%) + BC (2%) + MT (100 µM)

Overall, we found a significant (*p* ≤ 0.05) difference among treatments for gene expression (Fig. 5). Under Pb and MPs stress, plant receiving combined BC + MT application showed significant (*p* ≤ 0.05) increase in *OsAPx6*, *OsCAT*, *OsPOD*, and *OsSOD* by 27.86%, 34.14%, 33.92% and 43.13% respectively (Table 1). Overall, co-applied BC and MT resulted in the maximum enhancement in *OsASMT*1 expression, reaching 32.53% while *OsP5CS* expression reached to 29% (Fig. 5). Biochar combined with MT suppressed the expression of Pb uptake-related genes, (*OsHMA9* and *OsNRAMP5*), by 28.05% and 29.97% (Fig. 5).

### Tissue nutrient and lead accumulation and soil properties

**Fig. 6:**
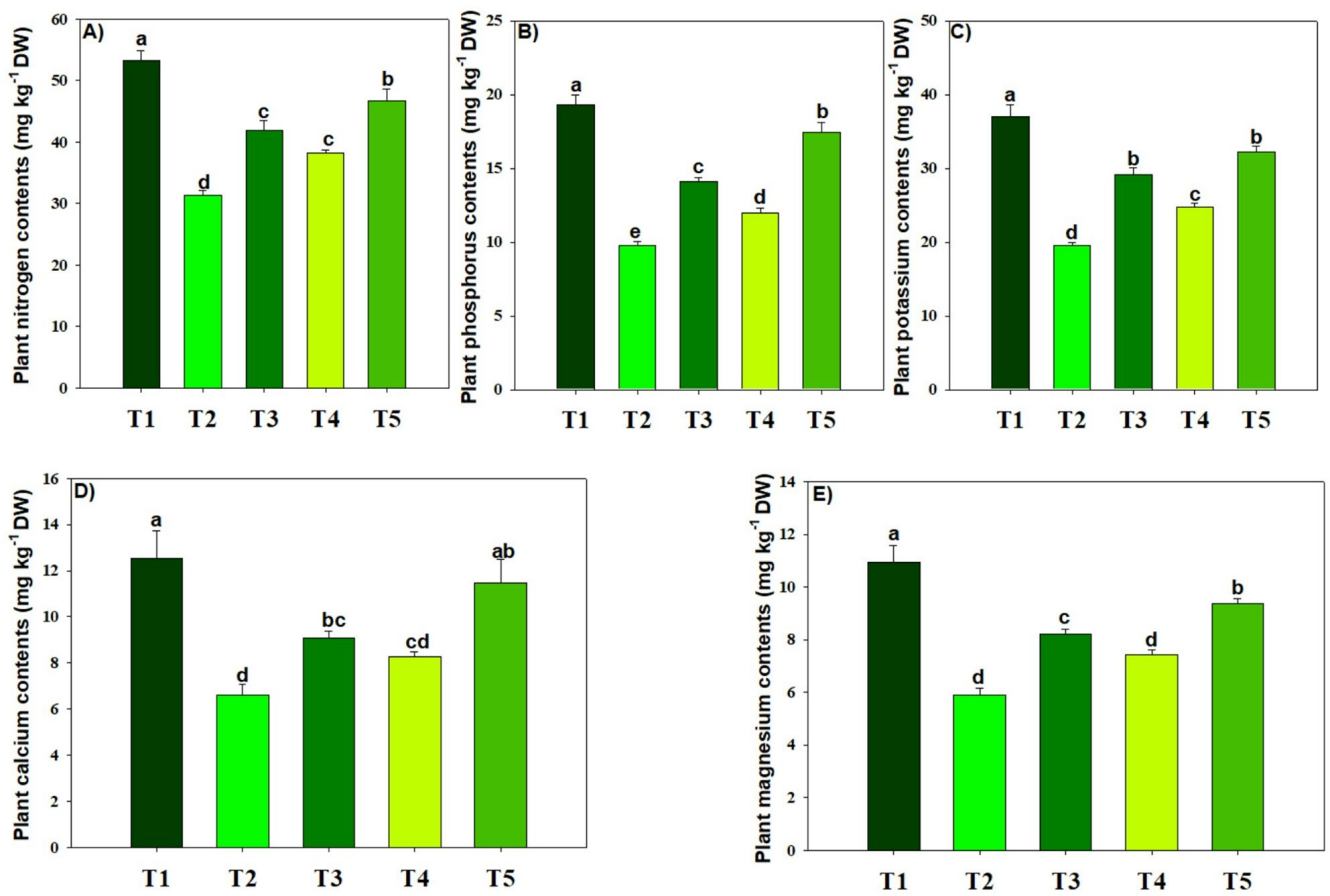
The effects of biochar and foliar applied melatonin on nutrient accumulation in rice plans (**a**-**e**) grown in combined lead and micro-plastics contaminated soil. The data is mean of three replicates (*n* = 3) and same letters showing non-significant difference as determined by HSD test (0.05 probability level). T_1_: control, T_2_: Pb (250 mg kg^− 1^) + MPs (1%), T_3_: Pb (250 mg kg^− 1^) + MPs (1%) + BC (2%), T_4_: Pb (250 mg kg^− 1^) + MPs (1%) + MT (100 µM) and T_5_: Pb (250 mg kg^− 1^) + MPs (1%) + BC (2%) + MT (100 µM)

**Fig. 7:**
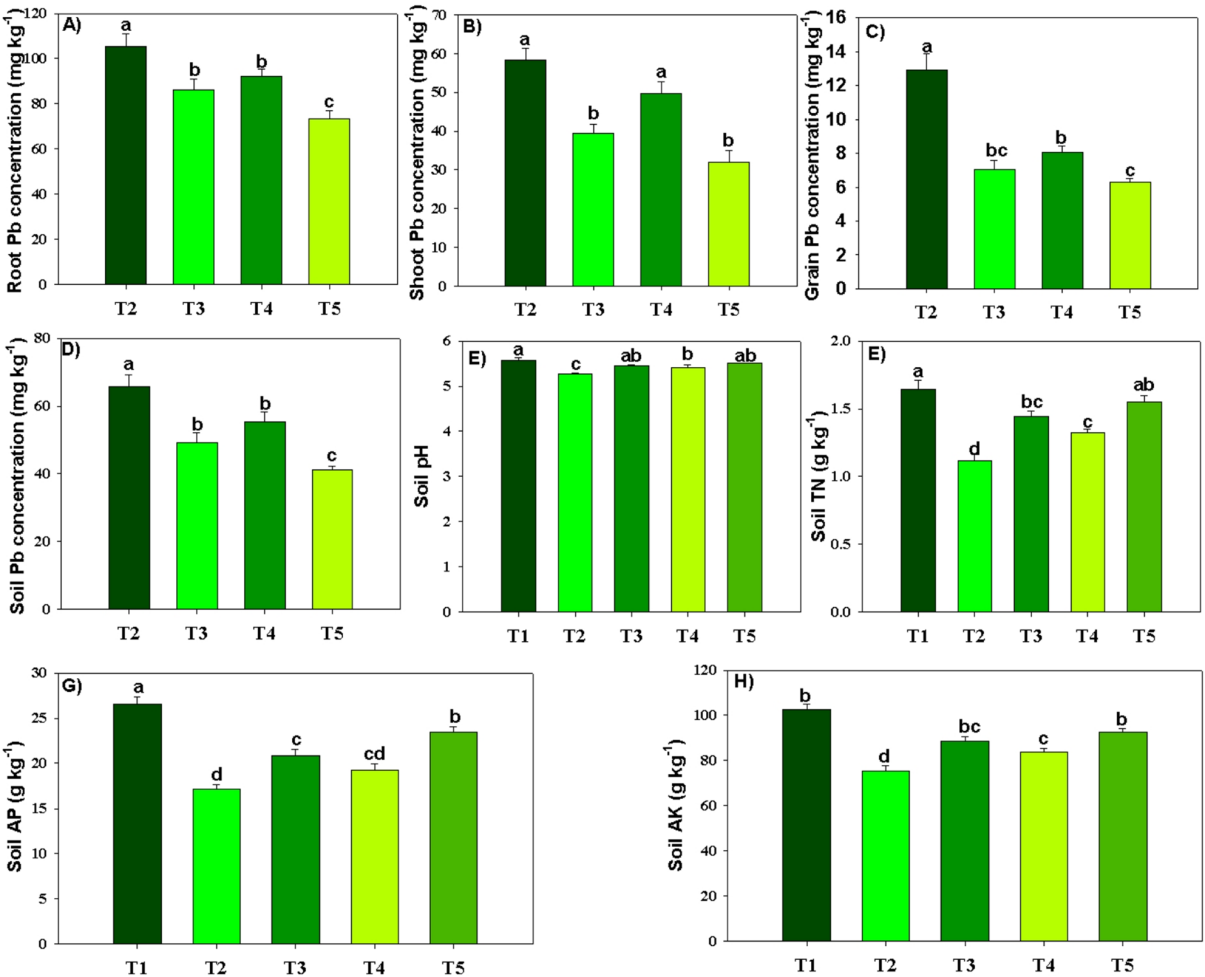
The effects of biochar and foliar applied melatonin on Pb accumulation in roots and shoots (**a**-**c**), soil Pb concentration (**d**), soil pH (**e**) and soil nutrients availability (**f**-**h**) after harvesting rice from combined lead and micro-plastics contaminated soil. The data is mean of three replicates (*n* = 3) and same letters showing non-significant difference as determined by HSD test (0.05 probability level). T_1_: control, T_2_: Pb (250 mg kg^− 1^) + MPs (1%), T_3_: Pb (250 mg kg^− 1^) + MPs (1%) + BC (2%), T_4_: Pb (250 mg kg^− 1^) + MPs (1%) + MT (100 µM) and T_5_: Pb (250 mg kg^− 1^) + MPs (1%) + BC (2%) + MT (100 µM). TN: total nitrogen, AP: available phosphorus, AK: available potassium

We observed that Pb + MPs reduced the N, P, K, Ca, and Mg accretion in rice plants by 41.31%, 49.53%, 47.17%, 47.28% and 54.92% respectively (Fig. 6). Conversely, BC and MT mitigated these adversities and enhanced the nutrient accretion in rice plants facing the MPs toxicity (Fig. 6). Of all the treatments, interactive use of BC and MT significantly (*p* ≤ 0.05) enhanced the N, P, K, Ca, and Mg accretion in rice plants by 32.96%, 44.06%, 39.42%, 42.56% and 37.13% respectively, under Pb and MPs stress (Fig. 6). Under Pb + MPs stress; maximum reduction in roots (30.45%), shoot (45.32), and grain Pb (59.01%), concentration was noted with BC + MT (Fig. 7). Lead and MPs decreased the soil pH by 5.56% after harvesting the rice crop (Fig. 7). Biochar + MT enhanced the soil pH contaminated with Pb and MPs. The combination of lead and MPs reduced soil N, P, and K by 32.42%, 35.31%, and 26.71%, respectively (Fig. 7). Nevertheless, BC + MT increased soil TN, AP, and AK availability (Fig. 7). Of all treatments, co-applied BC + MT significantly (*p* ≤ 0.05) increased TN, AP, and AK concentration by 28.19%, 26.73% and 18.81% respectively, (Fig. 7). The soil Pb availability was augmented under Pb and MPs-polluted soil, but BC and MT curbed this increase and decreased Pb availability by 37.18% (Fig. 7).

### Trait interrelationships

**Fig. 8:**
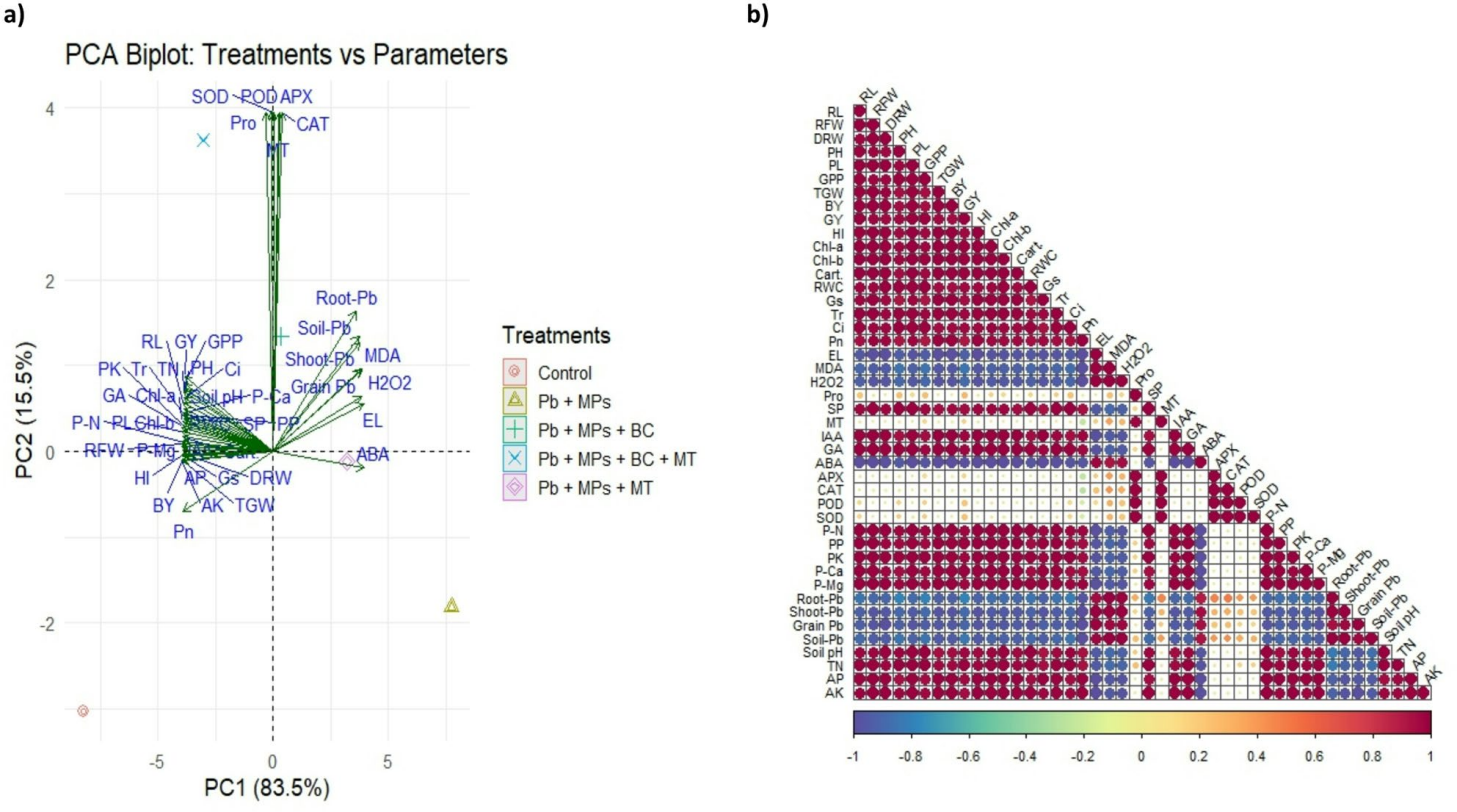
Principle component (**a**) and correlation analysis (**b**) for the impact of different treatments on studied traits. RL: root length, RFW: root fresh weight, RDW: root dry weight, PH: plant height, PL: panicle length, GPP: grains/panicle, TGW: 1000 grain weight, BY: biological yield, GY: grain yield, HI: harvest index. Chl: chlorophyll, cart. carotenoids, RWC: relative water contents, Gs: stomatal conductance, Tr: transpiration rate, Pn: photosynthetic rate, Ci: intercellular carbon dioxide, EL electrolyte leakage, MDA: malondialdehyde, H_2_O_2_: hydrogen peroxide, Pro: proline, SP: soluble proteins, MT: melatonin, IAA: indole aceptic acid, GA: gibberellins, ABA: abscisic acid, APX: ascorbate peroxidase, POD: peroxidase, CAT: catalase, SOD: superoxide dismutase, PN: plant nitrogen, PP: plant phosphrous, PK: plant potassium, P-Ca: plant calcium, P-mg: plant magnesium. TN: total nitrogen, AP: available phosphorus, AK: available potassium

The data were subjected to PCA and correlation analysis to determine the relationship between the studied traits (Fig. 8). First PCA showed a contribution of 83.3% while the second PCA showed a contribution of 15.8%. The results showed that control treatment observed on the negative side of PC1, showing lower stress and higher growth. The other treatments were associated with oxidative markers and Pb accumulation, showing an inhibitory effect on plant growth. The treatments receiving BC and MT were positioned on the positive side, showing the mitigation in stress and an increase in rice growth. There was a positive linking between, antioxidants, hormones and physiological traits suggesting the coordinated response to counteract Pb + MPs stress (Fig. 8). The negative linking between oxidative markers, Pb and growth traits, indicating that MPs and Pb mediated oxidative stress suppressed rice growth. Proline and antioxidants showed a positive association, emphasizing the role of proline in increasing the antioxidant activities and maintaining the osmotic adjustments. Additionally, Pb accumulation in plant showed a negative relationship with nutrients showing adverse impacts of Pb on nutrient uptake (Fig. 8).

## Discussion

Microplastic pollution is becoming a serious threat to agricultural productivity [55]. We observed that Pb and MPs, individually and in combination, significantly decreased rice growth and yield, a trend consistent with previous findings [25, 56]. The observed reduction in growth occurred through a series of underlying mechanisms. The decrease in plant growth was linked with inhibited nutrient uptake (N, P, and K). Lead is known to compete with nutrient ions [57], while MPs damage the cells of root epidermis [58], and compromise the ability of roots for water and nutrient absorption [26, 56]. Therefore, this decrease in nutrient uptake caused a marked reduction in rice growth. Besides this, MPs also contain a higher carbon to hydrogen ratio, alter the soil structure, microbial activities, and root proliferation, which further imped plant growth [59]. The observed decrease in rice growth was also associated with impaired photosynthetic efficiency. This decrease in photosynthetic capacity was linked with a combination of phytotoxicity induced by Pb and MPs. Lead stress caused oxidative damages by increasing ROS, which damages the photosynthetic apparatus [21], while MPs owing to their physical presence, induce oxidative stress, which downregulates plant metabolism, such as photosynthetic pigments and hormone production [60, 61]. Therefore, the subsequent reduction in assimilates productions explains the decrease in rice yield (Table 1). It is also worth mentioning that micro-plastics impacts are not universally negative, with some studies reporting an increase in plant growth in response to MPs [62]. These variations likely arise from MPs’ type, size, polymer type, and duration of exposure [63] and their effects on soil properties [64]. 

Biochar and MT synergistically alleviated the Pb and MPs toxicity and improved rice growth and productivity (Table 1). These protective effects were achieved through a different interconnected mechanism. The primary underlying mechanism was BC + MT mediated improved root growth. Biochar improves soil porosity, CEC, and water retention, which provides a favorable environment for root growth [25, 65]. Concurrently, MT improved the root elongation and growth by improving the IAA synthesis (Fig. 4). The robust root system may have ensured efficient nutrient and water uptake, therefore supporting the plant growth [66, 67]. The hormonal balance play a critical role in plants; however, Pb + MPs severely disrupted this balance as noted by a decrease in IAA and GA synthesis, with an increase in ABA accumulation. The decrease in IAA and GA synthesis also explains the observed growth reduction. Biochar + MT significantly enhanced IAA and GA synthesis while suppressing the ABA production. Melatonin modulates IAA and GA pathways, while BC improves nutrient which provide metabolic precursors for hormone synthesis [68]. This rebalance of hormonal synthesis favors cell division and plant growth in response to stress conditions. Photosynthetic pigments play an imperative role in biomass production [69]; however, Pb and MPs decreased chlorophyll synthesis via decreasing Mg availability and increasing ROS production [70–73]. Moreover, MPs cause surface damage to the thylakoid membranes and compromise the photosynthetic efficiency. This decrease in photosynthetic efficiency limits carbon assimilation and subsequent dry matter production [71]. Biochar + MT synergistically enhanced Mg uptake and reduced ROS production, which protected the photosynthetic apparatus, thereby ensuring better growth. Biochar and MT together provided greater stress alleviation than their individual application. The observed improvement in growth indicates an additive effect where BC improves soil health, while favors cellular activity.

Gas exchange traits provide insights into processes involved in carbon and water fluxes in the leaves [74]. Notably, Pb + MPs reduced leaf carbon and water exchange (Fig. 2), and led to down-regulation of Pn, Tr, Gs, and Ci (Fig. 2). This observed reduction in gas exchange traits is linked with the accumulation of MPs particles in the rice root zone and Pb accretion in roots and aboveground parts. The observed effect could be due to the adherence of MPs to root surfaces, resulting in pore blockage, altered root structural integrity, and disturbed water homeostasis, collectively suppressing water uptake [75]. This physical barrier decreases the root’s hydraulic conductivity, thereby limiting water uptake and transport to aerial parts. The resulting water deficiency triggers the stomatal closure, which consequently decreases Tr and limits CO_2_ diffusion, ultimately constraining the Pb [76]. Moreover, Pb accumulation in roots exacerbates these effects through different mechanisms. For instance, Pb in roots impairs nutrient and water transportation by disturbing aquaporin function and damaging root cell membranes. In leaves, Pb directly interferes with the photosynthetic apparatus, damaging the electron transport chain, chloroplast structure, and RuBisCO activity [77, 78]. Hence, the combination of MPs mediate damage to roots and Pb-induced cellular toxicity creates a combined stress, which limits the photosynthetic efficiency.

Biochar + MT enhanced Chl contents and gas exchange traits. The increase was possibly linked with decreased MPs entry to roots by BC and an increase in antioxidants synthesis and subsequent decrease in ROS production by BC + MT (Fig. 3). Magnesium functions as a structural element in Chl formation, and Pb + MPs decreased Mg acceration in rice seedlings (Fig. 6). Biochar + MT synergistically enhanced the Mg uptake and accumulation, which provides support for the notion that increased Mg accumulation enhanced the chlorophyll synthesis [79, 80]. We also witnessed that BC + MT enhanced the root growth (Table 1), and this increase in root growth favors the minerals and water uptake, leading to better Chl contents [81]. Lead and MPs decreased the leaf RWC, which was linked with decreased root growth (Table 1) and cell turgor pressures and root hydraulic conductivity. We found that BC + MT efficiently maintained better root growth by alleviating the Pb and MPs toxicity, which enhanced the water uptake and resulted in better RWC [28].

Lead and MPs significantly enhanced the ROS production, making the plants to highly vulnerable to oxidative damage. The increase in MDA production damages the membrane via increasing the lipid peroxidation [82, 83]. Antioxidant enzymes are a robust defense system used by plants facing stress conditions [84]. We found that the activity of antioxidants showed a little increase in response to Pb and MPs’ toxicity, providing evidence that plants enhanced antioxidant defense to counter Pb + MPs’ toxicity. The increase in antioxidant activity after Pb and MPs exposure is in accordance with previous studies reporting an increase in antioxidants under MPs [25, 55, 60]. Biochar and MT synergistically enhanced the antioxidants, which helped in decreasing the Pb and MPs-induced damage. Biochar and MT effectively up-regulated the antioxidant genes (*OsAPx6*, *OsCAT*, *OsPOD*, and *OsSOD*). This can be ascribed to the role of MT as a direct scavenger of ROS and signaling molecule that activates antioxidant defense at the transcriptional level. Melatonin also stimulates the transcription factors binding the promoter region of antioxidants genes, and it also interacts with nitric oxide signaling pathways to enhance their expression. Thus, this molecular cascade led to asignificant increase in antioxidants activities (41.17–52.55%) and the subsequent decrease in MDA and H₂O₂ production. Biochar adsorb Pb, hence contributing to a decrease in Pb availability. This decrease in Pb availability may have resulted in increased antioxidant activities. Moreover, the release of biopolymers and organic materials from BC also favors antioxidants activities [85], hence, the combination of BC + MT showed much better results in increasing antioxidant activity.

Biochar also reduces the MPs transportation into aerial parts by increasing the MPs adsorption, which leads to a reduction in H_2_O_2_ production [72, 86]. We also found that BC and MT enhanced the endogenous MT synthesis (Fig. 4), which improves the antioxidant defense and helps in mitigating the ROS and MDA production [28]. Another potential reason is that BC, owing to its porous structure and large surface area, may have protected MT from degradation, thereby enhancing its stability in the soil. Biochar improves soil properties and root growth and development. Therefore, this improved root system possibly helped in taking the MT from the soil solution, hence contributing to better growth and stress resilience. *OsASMT1* encodes the enzymes that catalyze the final step of MT biosynthesis [87]. The promoter region of *OsASMT1* contains stress responsive elements, which explains its up-regulation in response to MPs and Pb stress. The foliar spray of MT further enhanced the expression of *OsASMT1*, which may have created the positive feedback loop which in turned enhanced endogenous MT levels. Thus, the observed up-regulation in *OsASMT1* by BC + MT application shows a key mechanism mediated by combined amendment to enhance Pb + MPs tolerance in rice.

Hormonal signaling governs plant growth and stress adaptation. We found that Pb + MPs disturbed this balance by increasing ABA production while decreasing IAA and GA production. This shift in hormonal balance explains the decrease in plant growth. The synthesis of SP was also decreased under Pb + MPs stress, indicating an impaired N metabolism. Lead and MPs reduced the N uptake and availability (Figs. 6 and 7), which may have limited the amino acid substrates needed for protein synthesis. Therefore, this N limitation leads to a serious decrease in SP synthesis. Biochar + MT restored the hormonal balance by increasing IAA and GA synthesis, while decreasing ABA synthesis. This increase in IAA and GA synthesis is possibly linked with MT mediated increase in gene expression of these hormones and improvement in soil properties after BC application, stimulating the hormonal pathways. Biochar and MT also improved the N availability, which may provide the necessary substrates for protein synthesis, contributing to improved SP synthesis in response to Pb + MPs stress.

Microplastics have a large surface area, and they interact with Pb through charged and polar regions; therefore, increasing the Pb availability in soil [88, 89]. Further, the decrease in soil pH, likely play the role in increasing Pb mobility [90]. Additionally, the increased mobility and uptake of Pb by rice plants also resulted from MPs occupying the soil sorption sites, which kept the mobilization of Pb and prevented its stabilization [91]. Biochar and MT decreased Pb accretion though a set of mechanisms. The alkaline nature of BC increased the soil pH, which may have caused Pb immobilization, thus decreasing its availability to plants. Notably, BC + MT also down-regulated the genes such as *OsNRAMP5* and *OsHMA9* involved in Pb uptake. *OsNRAMP5* works as an influx transporter in root cells [92], therefore its up-regulation under Pb stress led to initial Pb uptake from soil solutions and accumulation in rice plants. Biochar and MT down-regulated the expression of *OsNRAMP5*, which decreased the Pb influx into the plant tissues, thus diminishing the Pb accumulation in plant tissues. *OsHMA9* is a P1B-type ATPase efflux protein that transports metal out of cells [93]. The observed down-regulation of *OsHMA9* after BC + MT application indicates that reduced cellular burden of Pb owing to its diminished uptake, thus decreasing the demand of efflux activity. Therefore, this down-regulation of influx and efflux transporters decreased the Pb accumulation in rice plants.

Lead and MPs decreased the soil pH (Fig. 7), aligning with previous studies of Janczak et al. [94], while in contrast with the findings of Gao et al. [78] and Wang et al. [95]. Microplastics release H⁺ and lactic acid, which microbes transform into acetic acid and other metabolites, thereby lowering soil pH [96]. The availability of soil nutrients was also decreased in Pb and MPs-contaminated soil, providing evidence that they negatively affected the soil N and P cycling, therefore decreasing the availability of these nutrients. Compared to the control, BC increased the soil pH, while MT showed a minor change in soil pH. Biochar used in this study contained alkaline substances, and it also had a pH of 9.63, which contributed to an increase in soil pH [97]. The large surface area of BC also absorbs acid ions (H^+^), which reduces their activity, thereby increasing the soil pH [98]. The decomposition of BC derived organic matter also contributes to an increase in soil pH via the release of alkaline compounds, including carbonates, biocharbonates, and hydroxide [98]. Moreover, BC + MT enhanced soil nutrient availability, owing to input of nutrients from BC and improvement in soil nutrients availability [99]. Biochar + MT synergistically enhanced root growth, which improves the nutrient uptake from soil, therefore, leading to better accumulation of nutrients in rice plants under Pb and MPs polluted soil [100]. It is important to mention that microplastics can degrade over time, potentially altering their interaction with heavy metals such as Pb. The present study did not monitor the aging, which is a limitation of our study. Therefore, it is suggested that in future studies, the aging and degradation of MPs should be studied over time, as these factors play a critical role in modulating their interaction with Pb. The application of 2% BC is feasible owing to its long-term benefits, such as improving soil health and crop productivity. Nevertheless, application of MT (100µM) may need optimization for determine its cost-effectiveness. Additionally, the economic feasibility of using 2% BC and MT (100µM) must be tested in increasing crop productivity and reducing the contamination risks.

## Conclusion

Lead and microplastics decreased the above and below ground rice growth by impairing the plant functioning, decreasing soil pH, nutrient availability, and increasing lead accumulation. In contrast, biochar and melatonin enhanced rice yield by increasing soil pH, nutrient availability, antioxidant activity, osmolytes synthesis, gene expression, and maintenance of hormonal balance. Therefore, integrated biochar and melatonin can alleviate lead + microplastics by improving soil health and plant functioning. However, long-term impacts of the biochar and melatonin must be studied in field conditions. The metabolomics and transcriptomic studies are also needed to study underlying mechanisms involved in counteracting lead and micro-plastics toxicity. The future studies should also conduct the sequential extraction procedure to analyze lead fractions. This will provide mechanistic insights if biochar and melatonin cause lead immobilization by adsorption or precipitation. The present study used the 1% micro-plastics rate to facilitate comparisons with previous studies. However, this rate is high compared to real-world conditions. Therefore, future studies should include a wide range of microplastic concentrations, such as 0.001%, 0.01%, and 0.1%, to measure the dose-dependent impacts of microplastics on lead toxicity and interactions. 

## Electronic Supplementary Material

Below is the link to the electronic supplementary material.

Supplementary Material 1.

## Data Availability

The data is contained within the article. Further correspondence can be made to the corresponding author.

## References

[1] Wang C, Wu B, Jiang K, Wei M, Wang S. Effects of different concentrations and types of Cu and Pb on soil N-fixing bacterial communities in the wheat rhizosphere. Appl Soil Ecol. 2019;144:51–9.

[2] PlasticsEurope. Plastics – the Facts 2024: An analysis of European plastics production, demand and waste data. Brussels: PlasticsEurope. 2024 [ Available from:` https://plasticseurope.org/knowledge-hub/plastics-the-fast-facts-2024 ]. Cited 26 Jan 2026.

[3] Corradini F, Meza P, Eguiluz R, Casado F, Huerta-Lwanga E, Geissen V. Evidence of microplastic accumulation in agricultural soils from sewage sludge disposal. Sci Total Environ. 2019;671:411–20. 30933797 10.1016/j.scitotenv.2019.03.368

[4] Zhao S, Zhang Q, Chen X, Huang Q, Li H, Siddique KHM. Biochar counteracts the negative effects of microplastics on physiological and biochemical characteristics and leaf metabolism in Zea mays L. J Hazard Mater. 2025;496:139355. 40749652 10.1016/j.jhazmat.2025.139355

[5] Ren X, Tang J, Wang L, Liu Q. Microplastics in soil-plant system: effects of nano/microplastics on plant photosynthesis, rhizosphere microbes and soil properties in soil with different residues. Plant Soil. 2021;462:561–76.

[6] Wu C, Ma Y, Wang D, Shan Y, Song X, Hu H, Ma Y. Integrated microbiology and metabolomics analysis reveal plastic mulch film residue affects soil microorganisms and their metabolic functions. J Hazard Mater. 2022;423:127258. 10.1016/j.jhazmat.2021.127258. 34844367 10.1016/j.jhazmat.2021.127258

[7] Yu Y, Li J, Song Y, Zhang Z, Yu S, Xu M, Zhao Y. Stimulation versus inhibition: the effect of microplastics on pak choi growth. Appl Soil Ecol. 2021;177:104505.

[8] Feng X, Sun Y, Zhang S, Wang F. Ecological effects of microplastics on soil-plant systems. Acta Pedol Sin. 2021;58(2):299–313.

[9] Yao Y, Wang L, Pan S, Li G, Liu H, Xiu W, Gong L, Zhao J, Zhang G, Yang D. Can microplastics mediate soil properties, plant growth and carbon/nitrogen turnover in the terrestrial ecosystem? Ecosyst Health Sustain. 2022;8(1):2133638.

[10] Mondal NK, Kundu S, Debnath P, Mondal A, Sen K. Effects of polyethylene terephthalate microplastic on germination, biochemistry and phytotoxicity of Cicer arietinum L. and cytotoxicity study on Allium cepa L. Environ Toxicol Pharmacol. 2022;94:103908. 35709962 10.1016/j.etap.2022.103908

[11] Wang C, Bian J, Min W, Zhao X. Effects of two PBAT/PLA biodegradable mulch film fragments on soil dissolved organic carbon and nitrogen and their phytotoxicity. Asian J Ecotoxicol. 2022;5:465–74.

[12] Fei Y, Huang S, Zhang H, Tong Y, Wen D, Xia X, Wang H, Luo Y, Barcelo D. Response of soil enzyme activities and bacterial communities to the accumulation of microplastics in an acid cropped soil. Sci Total Environ. 2020;707:13563. 10.1016/j.scitotenv.2019.135634 31761364

[13] Jari S, Algethami, Muhammad I, Wasim J, Eid H, Alosaimi, Muhammad KI. Efficacy of Fe-BC in enhancing growth, photosynthesis, nutrition, and alleviating the toxicity of Cd and Cr in rapeseed (*Brassica napus* L.): A tool for managing the environment and attaining sustainable agriculture. Environ Technol Innov. 2024;36:103789.

[14] Yang Z, Jiang L, Li X, Ji Q, Wang M, Zhang Y, Cheng Y, Zhang X, Li H, Feng C. Role of sludge biochar immobilized multifunctional microbiome in phytoremediation of lead-zinc composite pollution. Biochar. 2025;7:5.

[15] Irshad MK, Saleem S, Ansari JR, Noman A, Aqeel M, Alshahrani MO, Alotaibi MO, Irshad MS, Lee SS. An analysis of impact of soil co-pollution with lead and traditional/biodegradable microplastics on growth characteristics of maize (*Zea mays* L). J Environ Sci. 2025;1001:0742. 10.1016/j.jes.2025.09.022 42336526

[16] Aqeel M, Noman A, Nawaz S, Khalid N, Irshad MK, Iqbal MF, Alotaibi MO, Al-Mutairi M, Alshahrani MO, Alotaibi NM, Alzuaibr FM. Ecotype-specific responses of Typha domingensis to microplastics: Antioxidant defense and biochemical adaptations in wetland ecosystems. Environ Chem Ecotoxicol. 2025;7:1518–31.

[17] Khalid N, Aqeel M, Lee SY, Ejaz U, Noman A, Maqsood MF, Irshad MK, El-Sheikh MA. Association of microplastics with lead and cadmium in soil: land-use and temporal trends. J Environ Chem Engin. 2025;13:117067.

[18] Oh SJ, Irshad MK, Kang MW, Roh HS, Jeon Y, Lee SS. In-situ physical and chemical remediation of Cd and Pb contaminated mine soils cultivated with Chinese cabbage: A three-year field study. J Hazard Mat. 2023;459:132091. 10.1016/j.jhazmat.2023.132091 37515987

[19] Tian X, Wei X, Qin L, Zhang Y, Xiang Q, Zhao K, Yu X, Chen Q, Zhang L, Penttinen P, Gu Y. Buckwheat responds to co-exposure to PLA microplastics and Pb by regulating the synthesis of unsaturated fatty acids and jasmonates. J Hazard Mater. 2025;486:137066. 39764956 10.1016/j.jhazmat.2024.137066

[20] Jin HY, Xue ZH, Farooq U, Wei ZD, Wang L, Liu LX. Impact of polypropylene microplastics and lead on wheat germination and seedling growth. Environ Poll Bioavail. 2025;37:2478198.

[21] Tian X, Weixie L, Wang S, Zhang Y, Xiang Q, Yu X, Zhao K, Zhang L, Penttinen P, Gu Y. Effect of polylactic acid microplastics and lead on the growth and physiological characteristics of buckwheat. Chemosphere. 2023;337:139356. 37379973 10.1016/j.chemosphere.2023.139356

[22] Schmidt HP, Kammann C, Hagemann N, Leifeld J, Bucheli TD, Sanchez-Monedero MA, Cayuela ML. Biochar in agriculture–A systematic review of 26 global meta-analyses. GCB Bioenergy. 2021;13:1708–30.

[23] Dominchin MF, Verdenelli RA, Berger MG, Aoki A, Meriles JM. Impact of N-fertilization and peanut shell biochar on soil microbial community structure and enzyme activities in a Typic Haplustoll under different management practices. Eur J Soil Biol. 2021;104:103298.

[24] Liu Y, Wen Y, Cai H, Song X, Wang X, Zhang Z. Stress of polyethylene and polylactic acid microplastics on pakchoi (*Brassica rapa* subsp. chinensis) and soil bacteria: Biochar mitigation. J Hazard Mater. 2025;487:137301. 39847935 10.1016/j.jhazmat.2025.137301

[25] Khan TA, Su Q, Guoqin H, Zhixuan D, Noor MA, Asseri TA, Hassan DMU. Integrative Biochar and melatonin application mitigates lead toxicity in rice by modulating antioxidant activities, iron paque formation and downregulating the expression of metal uptake genes. Front Plant Sci. 2025;16:1609825. 40678561 10.3389/fpls.2025.1609825 PMC12267010

[26] Yang Y, Liu L, Xiong H, Wang T, Yang J, Wang W, Al-Khalaf AA, Wang Z, Ahmed W. Biochar and trehalose co-Application: a sustainable strategy for alleviating lead toxicity in rice. Plants. 2025;14(6):878. 40265793 10.3390/plants14060878 PMC11946277

[27] Emamverdian A, Ghorbani A, Pehlivan N, Alwahibi MS, Elshikh MS, Liu G, Li Y, Barker J, Zargar M, Chen M. Co-application of melatonin and zeolite boost bamboo tolerance under cadmium by enhancing antioxidant capacity, osmolyte accumulation, plant nutrient availability, and decreasing cadmium absorption. Sci Hortic. 2023;322:112433.

[28] Noor MA, Hassan MU, Khan TA, Zhou B, Huang G. Biochar and Melatonin Partnership Mitigates Arsenic Toxicity in Rice by Modulating Antioxidant Defense, Phytochelatin Synthesis, and Down-Regulating the Transporters Involved in Arsenic Uptake. Plants. 2025;14(15):2453. 10.3390/plants14152453 PMC12349230 40805803

[29] Ghorbani A, Emamverdian A, Pishkar L, Chashmi KA, Salavati J, Zargar M, Chen M. Melatonin-mediated nitric oxide signaling enhances adaptation of tomato plants to aluminum stress. South Afr J Bot. 2023;162:443–50.

[30] Li X, Ahammed GJ, Zhang X-N, Zhang L, Yan P, Zhang L-P, Fu J-Y, Han W-Y. Melatonin-mediated regulation of anthocyanin biosynthesis and antioxidant defense confer tolerance to arsenic stress in *Camellia sinensis* L. J Hazard Mater. 2021;403:123922. 33264973 10.1016/j.jhazmat.2020.123922

[31] Li M, Liu Y, Xu G, Wang Y, Yu Y. Impacts of polyethylene microplastics on bioavailability and toxicity of metals in soil. Science of the Total Environment. 2021;760:144037. 10.1016/j.scitotenv.2020.144037 33348149

[32] Kim SK, Kim JS, Lee H, Lee HJ. Abundance and characteristics of microplastics in soils with different agricultural practices: Importance of sources with internal origin and environmental fate. J Hazard Mater. 2021;403:123997. 33265033 10.1016/j.jhazmat.2020.123997

[33] Liu Y, Guo R, Zhang S, Sun Y, Wang F. Uptake and translocation of nano/microplastics by rice seedlings: Evidence from a hydroponic experiment. J Hazard Mater. 2021;403:123997. 34332487 10.1016/j.jhazmat.2021.126700

[34] Zhang Z, Zhao L, Jin Q, Luo Q, He H. Combined contamination of microplastic and antibiotic alters the composition of microbial community and metabolism in wheat and maize rhizosphere soil. J Hazard Mater. 2024;473:134618. 38761764 10.1016/j.jhazmat.2024.134618

[35] Harter J, Krause HM, Schuettler S, Ruser R, Fromme M, Scholten T, Kappler A, Behrens S. Linking N_2_O emissions from biochar-amended soil to the structure and function of the N-cycling microbial community. ISME J. 2014;8(3):660–74. 24067258 10.1038/ismej.2013.160 PMC3930306

[36] Shar AG, Hussain S, Junaid MB, Hussan MU, Zulfiqar U, AlGarawi AM, Popielec R, Zhang L, Artyszak A. Melatonin ameliorates cadmium toxicity in tobacco seedlings by depriving its bioaccumulation, enhancing photosynthetic activity and antioxidant gene expression. Plants 2024;13;3049. 10.3390/plants13213049 PMC11548336 39519967

[37] Song C, Manzoor MA, Mao D, Ren X, Zhang W, Zhang Y. Photosynthetic machinery and antioxidant enzymes system regulation confers cadmium stress tolerance to tomato seedlings pretreated with melatonin. Sci Hortic. 2024;323:112550.

[38] Wang K, He J, Gao Y, Han K, Liu J, Wang Y. Exogenous melatonin improved the growth and development of naked oat seedlings under cadmium stress. Environ Sci Poll Res. 2022;29:88109–18. 10.1007/s11356-022-21798-3 35821327

[39] Arnon DI. Copper enzymes in isolated chloroplasts. Polyphenoloxidase in *Beta Vulgaris*. Plant Physiol. 1949;24:1–15. 16654194 10.1104/pp.24.1.1 PMC437905

[40] Hodges DM, DeLong JM, Forney CF, Prange RK. Improving the thiobarbituric acid-reactive-substances assay for estimating lipid peroxidation in plant tissues containing anthocyanin and other interfering compounds. Planta. 1999;207:604–11. 10.1007/s00425-017-2699-3 28456836

[41] Velikova V, Yordanov I, Edreva A. Oxidative stress and some antioxidant systems in acid rain-treated bean plants: Protective role of exogenous polyamines. Plant Sci. 2000;151:59–66.

[42] Valentovic P, Luxova M, Kolarovic L, Gasparikova O. Effect of osmotic stress on compatible solutes content, membrane stability and water relations in two maize cultivars. Plant Soil Environ. 2006;52:184.

[43] Chattha MU, Hassan MUU, Khan I, Nawaz M, Shah AN, Sattar A, Hashem M, Alamri S, Aslam MT, Alhaithloul HAS, Hassan MU, Qari SH. Hydrogen peroxide priming alleviates salinity induced toxic effect in maize by improving antioxidant defense system, ionic homeostasis, photosynthetic efficiency and hormonal crosstalk. Mol Biol Rep. 2022;49(6):5611–24. 35618939 10.1007/s11033-022-07535-6

[44] Bradford M. A Rapid and sensitive method for the quantitation of microgram quantities of protein utilizing the principle of protein-dye binding. Anal Biochem. 1976;72(12):248–54. 942051 10.1016/0003-2697(76)90527-3

[45] Bates LS, Waldren RA, Teare I. Rapid determination of free proline for water-stress studies. Plant Soil. 1973;39:205–7.

[46] Byeon Y, Back K. An increase in melatonin in transgenic rice causes pleiotropic phenotypes, including enhanced seedling growth, delayed flowering, and low grain yield. J Pineal Res. 2014;56(4):408–14. 24571270 10.1111/jpi.12129

[47] Koshita Y, Takahara T, Ogata T, Goto A. Involvement of endogenous plant hormones (IAA, ABA, GAs) in leaves and flower bud formation of satsuma mandarin (*Citrus unshiu* Marc). Sci Hortic. 1999;79:185–94.

[48] Zhang W, Zhang F, Raziuddin R, Gong H, Yang Z, Lu L, Ye Q, Zhou W. Effects of 5-aminolevulinic acid on oilseed rape seedling growth under herbicide toxicity stress. J Plant Growth Regul. 2008;27:159–69.

[49] Xia XJ, Wang YJ, Zhou YH, Tao Y, Mao WH, Shi K, Asami T, Chen Z, Yu JQ. Reactive oxygen species are involved in brassinosteroid-induced stress tolerance in cucumber. Plant Physiol. 2009;150(2):801–14. 19386805 10.1104/pp.109.138230 PMC2689980

[50] Aebi H. Catalase in vitro. Methods Enzymol. 1984;105:121–6. 6727660 10.1016/s0076-6879(84)05016-3

[51] Livak KJ, Schmittgen TD. Analysis of relative gene expression data using real-time quantitative PCR and the 2^–∆∆CT^ Method. Methods. 2001;25:402–8. 11846609 10.1006/meth.2001.1262

[52] Bao SD. Soil Agricultural Chemical Analysis. 3rd ed. Beijing: China Agricultural Press; 2000. ISBN: 9787109066441.

[53] Olsen SR, Sommers LE. Phosphorus. In: Page AL, Miller RH, Keeney DR, editors. Methods of Soil Analysis: Part 2 Chemical and Microbiological Properties. 2nd ed. Madison, WI: American Society of Agronomy and Soil Science Society of America; 1982. pp. 403–30.

[54] Helmke PA, Sparks DL, Lithium. Sodium, Potassium, Rubidium, and Cesium. In: Sparks DL, Page AL, Helmke PA, et al. editors. Methods of Soil Analysis, Part 3: Chemical Methods. 5th ed. Madison, WI: Soil Science Society of America and American Society of Agronomy; 2018. pp. 551–74.

[55] Li W, Wufuer R, Duo J, Wang S, Luo Y, Zhang D, Pan X. Microplastics in agricultural soils: Extraction and characterization after different periods of polythene film mulching in an arid region. Sci Total Environ. 2020;749:141420. 32836118 10.1016/j.scitotenv.2020.141420

[56] Liu Y, Xu F, Ding L, Zhang G, Bai B, Han Y, Xiao L, Song Y, Li Y, Wan S, Li G. Microplastics reduce nitrogen uptake in peanut plants by damaging root cells and impairing soil nitrogen cycling. J Hazard Mater. 2023;443:130384. 36444071 10.1016/j.jhazmat.2022.130384

[57] Lian Y, Shi R, Liu J, Zeb A, Wang Q, Wang J, Yu M, Li J, Zheng Z, Ali N, Bao Y, Liu W. Effects of polystyrene, polyethylene, and polypropylene microplastics on the soil-rhizosphere-plant system: phytotoxicity, enzyme activity, and microbial community. J Hazard Mater. 2024;465:133417. 38183945 10.1016/j.jhazmat.2023.133417

[58] Meng F, Harkes P, van Steenbrugge JJM, Geissen V. Effects of microplastics on common bean rhizosphere bacterial communities. Appl Soil Ecol. 2023;181:104649.

[59] Xiao X, Chen Z, Chen B. H/C atomic ratio as a smart linkage between pyrolytic temperatures, aromatic clusters and sorption properties of biochars derived from diverse precursory materials. Sci Rep. 2016;6:22644. 26940984 10.1038/srep22644 PMC4778134

[60] Gao M, Xu Y, Liu Y, Wang S, Wang C, Dong Y, Song Z. Effect of polystyrene on di-butyl phthalate (DBP) bioavailability and DBP-induced phytotoxicity in lettuce. Environmental Pollution. 2021;268:115870. 10.1016/j.envpol.2020.115870 33120154

[61] Nuamzanei CU, Sk S, Kumar N, Borah B, Chikkaputtaiah C, Saikia R, Phukan T. Impact of polyvinyl chloride (PVC) microplastic on growth, photosynthesis and nutrient uptake of Solanum lycopersicum L. (Tomato). Environ Pollut. 2024;349:123994. 38636835 10.1016/j.envpol.2024.123994

[62] Khalid AR, Shah T, Asad M, Ali A, Samee E, Adnan F, Bhatti MF, Marhan S, Kammann CI, Haider G. Biochar alleviated the toxic effects of PVC microplastic in a soil-plant system by upregulating soil enzyme activities and microbial abundance. Environ Pollut. 2023;332:121810. 37201571 10.1016/j.envpol.2023.121810

[63] Baho DL, Bundschuh M, Futter MN. Microplastics in terrestrial ecosystems: moving beyond the state of the art to minimize the risk of ecological surprise. Glob Change Biol. 2021;27(17):3969–86. 10.1111/gcb.15724 34042229

[64] Qi Y, Beriot N, Gort G, Lwanga EH, Gooren H, Yang X, Geissen V. Impact of plastic mulch film debris on soil physicochemical and hydrological properties. Environ Pollut. 2020;266(Pt 3):115097. 32629308 10.1016/j.envpol.2020.115097

[65] Ren T, Feng H, Xu C, Xu Q, Fu B, Azwar E, Wei Y, Lam S, Liu G. Exogenous application and interaction of biochar with environmental factors for improving functional diversity of rhizosphere’s microbial community and health. Chemosphere. 2022;294:133710. 35074326 10.1016/j.chemosphere.2022.133710

[66] Joseph S, Cowie AL, Van Zwieten L, Bolan N, Budai A, Buss W, Cayuela ML, Graber ER, Ippolito JA, Kuzyakov Y, Luo Y, Ok YS, Palansooriya KN, Shepherd J, Stephens S, Weng Z, Lehmann J. How biochar works, and when it doesn’t: a review of mechanisms controlling soil and plant responses to biochar. Glob Change Biol Bioenergy. 2021;13:1731–64.

[67] Zhou J, Liu Y, Li B, Huang W, Qin J, Li H, Chen G. Hydrous zirconium oxide modified biochar for in situ remediation of arsenic contaminated agricultural soil. J Environ Chem Eng. 2022;10:108360.

[68] Jan R, Asif S, Asaf S, Lubna Du X-X, Park J-R, Nari K, Bhatta D, Lee I-j. Kim K-M. Melatonin alleviates arsenic (As) toxicity in rice plants via modulating antioxidant defense system and secondary metabolites and reducing oxidative stress. Environ Pollut. 2023;318:120868. 36526054 10.1016/j.envpol.2022.120868

[69] Xie H, Wei C, Wang W, Chen R, Cui L, Wang L, Chen D, Yu YL, Li B, Li YF. Screening the phytotoxicity of micro/nanoplastics through non-targeted metallomics with synchrotron radiation X-ray fluorescence and deep learning: Taking micro/nano polyethylene terephthalate as an example. J Hazard Mater. 2024;463:132886. 37913659 10.1016/j.jhazmat.2023.132886

[70] Shi R, Liu W, Lian Y, Wang X, Men S, Zeb A, Wang Q, Wang J, Li J, Zheng Z. Toxicity mechanisms of nanoplastics on crop growth, interference of phyllosphere microbes, and evidence for foliar penetration and translocation. Environ Sci Technol. 2024;58(2):1010–21. 37934921 10.1021/acs.est.3c03649

[71] Guzman-Tordecilla M, Pacheco-Bustos C, Coronado-Posada N, Pedrosa-Gomes M, Martinez-Burgos WJ, Mejía-Marchena R, Zorman-Marques R. Exploring the ecotoxicological impact of meropenem on Lemna minor: Growth, photosynthetic activity, and oxidative stress. Environ Res. 2024;258:119409. 38871272 10.1016/j.envres.2024.119409

[72] Li H, Song F, Song X, Zhu K, Lin Q, Zhang J, Ning G. Single and composite damage mechanisms of soil polyethylene/polyvinyl chloride microplastics to the photosynthetic performance of soybean (*Glycine max* [L.] merr). Front Plant Sci. 2023;13:1100291. 36743543 10.3389/fpls.2022.1100291 PMC9889878

[73] Yu H, Zhang X, Hu J, Peng J, Qu J. Ecotoxicity of polystyrene microplastics to submerged carnivorous Utricularia vulgaris plants in freshwater ecosystems. Environ Pollut. 2020;265:114830. Pt A). 32540562 10.1016/j.envpol.2020.114830

[74] Busch FA, Ainsworth EA, Amtmann A, Cavanagh AP, Driever SM, Ferguson JN, Kromdijk J, Lawson T, Leakey AD, Matthews JS, Meacham-Hensold K, Vath RL, Vialet-Chabrand S, Walker BJ, Papanatsiou M. A guide to photosynthetic gas exchange measurements: Fundamental principles, best practice and potential pitfalls. Plant Cell Environ. 2024;47(9):3344–64. 38321805 10.1111/pce.14815

[75] Gao B, Yao H, Li Y, Zhu Y. Microplastic addition alters the microbial community structure and stimulates soil carbon dioxide emissions in vegetablegrowing soil. Environ Toxicol Chem. 2021;40(2):352–65. 33105038 10.1002/etc.4916

[76] Ceccanti C, Davini A, Piccolo EL, Lauria G, Rossi V, Castiglione MR, Spanò C, Bottega S, Guidi L, Landi M. Polyethylene microplastics alter root functionality and affect strawberry plant physiology and fruit quality traits. J Hazard Mater. 2024;470:134164. 38583200 10.1016/j.jhazmat.2024.134164

[77] Mahamood MN, Zhu S, Noman A, Mahmood A, Ashraf S, Aqeel M, Ibrahim M, Ashraf S, Liew RK, Lam SS, Irshad MK. An assessment of the efficacy of biochar and zero-valent iron nanoparticles in reducing lead toxicity in wheat (Triticum aestivum L). Environ Pollut. 2023;319:120979. 36586554 10.1016/j.envpol.2022.120979

[78] Gao M, Liu Y, Song Z. Effects of polyethylene microplastic on the phytotoxicity of di-n-butyl phthalate in lettuce (*Lactuca sativa* L. var. ramosa Hort). Chemosphere. 2019;237:124482. 31398608 10.1016/j.chemosphere.2019.124482

[79] Hou Z, Tang Y, Li C, Lim K-J, Wang Z. The additive effect of biochar amendment and simulated nitrogen deposition stimulates the plant height, photosynthesis and accumulation of NPK in pecan (*Carya illinoinensis*) seedlings. AoB Plants. 2020;12(4):plaa035. 32850109 10.1093/aobpla/plaa035 PMC7441530

[80] Kamran M, Malik Z, Parveen A, Huang L, Riaz M, Bashir S, Mustafa A, Abbasi GH, Xue B, Ali U. Ameliorative effects of biochar on rapeseed (*Brassica napus* L.) growth and heavy metal immobilization in soil irrigated with untreated wastewater. J Plant Growth Regul. 2019;39:266–81.

[81] He Y, Yao Y, Ji Y, Deng J, Zhou G, Liu R, Shao J, Zhou L, Li N, Zhou X, Bai SH. Biochar amendment boosts photosynthesis and biomass in C_3_ but not C_4_ plants: A global synthesis. GCB Bioenergy. 2020;12:605–17.

[82] Zhang Y, Yao J, Yin K, Liu Z, Zhang Y, Deng C, Liu J, Zhang Y, Hou S, Zhang H, Yu D, Zhao N, Zhao R, Chen S. Populus euphratica phospholipase Dδ increases salt tolerance by regulating K+/Na + and ROS homeostasis in Arabidopsis. Int J Mol Sci. 2022;23(9):4911. Published 2022 Apr 28. 35563299 10.3390/ijms23094911 PMC9105705

[83] Ali S, Tyagi A, Bae H. ROS interplay between plant growth and stress biology: Challenges and future perspectives. Plant Physiol Biochem. 2023;203:108032. 37757722 10.1016/j.plaphy.2023.108032

[84] Li H, Dong S, Chen H, Wang Q, Zhang Y, Wang Y, Wang G. Deficit irrigation of reclaimed water relieves oat drought stress while controlling the risk of PAEs pollution in microplastics-polluted soil. J Environ Manage. 2024;366:121621. 38972188 10.1016/j.jenvman.2024.121621

[85] Liu ML, Lin Z, Ke XL, Fan XR, Joseph S, Taherymoosavi S, Liu XY, Bian RJ, Solaiman ZM, Li LQ, Pan GX. Rice seedling growth promotion by biochar varies with genotypes and application dosages. Front Plant Sci. 2021;12:580462. 34234791 10.3389/fpls.2021.580462 PMC8256797

[86] Li Jia, Yu YF, Cui M. Effects of biochar on the phytotoxicity of polyvinyl chloride microplastics in greenhouse soil. Plant Physiol Biochem. 2023;195:228–37. 36645927 10.1016/j.plaphy.2023.01.022

[87] Jassal PS, Paur TS, Wani AK. 2025. Phytomelatonin: A multifunctional molecule in plant defense and metabolism. Physiol Mol Plant Pathol. 141;102992.

[88] Li S, Guo J, Wang T, Gong L, Liu F, Brestic M, Liu S, Song F, Li X. Melatonin reduces nanoplastic uptake, translocation, and toxicity in wheat. J Pineal Res. 2021;71(3):e12761. 34392562 10.1111/jpi.12761

[89] Feng X, Wang Q, Sun Y, Zhang S, Wang F. Microplastics change soil properties, heavy metal availability and bacterial community in a Pb-Zn-contaminated soil. J Hazard Mater. 2022;424:127364. Pt A). 34879561 10.1016/j.jhazmat.2021.127364

[90] Yu Q, Gao B, Wu P, Chen M, He C, Zhang X. Effects of microplastics on the phytoremediation of Cd, Pb, and Zn contaminated soils by *Solanum photeinocarpum* and Lantana camara. Environ Res. 2023;231(Pt 3):116312. 37270082 10.1016/j.envres.2023.116312

[91] Zhang S, Han B, Sun Y, Wang F. Microplastics influence the adsorption and desorption characteristics of Cd in an agricultural soil. J Hazard Mater. 2020;388:121775. 31813687 10.1016/j.jhazmat.2019.121775

[92] Ishimaru Y, Takahashi R, Bashir K, Shimo H, Senoura T, Sugimoto K, Ono K, Yano M, Ishikawa S, Arao T, Nakanishi H. Characterizing the role of rice NRAMP5 in manganese, iron and cadmium transport. Sci Rep. 2012;2:286. 22368778 10.1038/srep00286 PMC3285952

[93] Lee S, Kim YY, Lee Y, An G. Rice P1B-type heavy-metal ATPase, OsHMA9, is a metal efflux protein. Plant physiology. 2007;145(3):831–42. 10.1104/pp.107.102236 PMC2048805 17827266

[94] Janczak K, Hrynkiewicz K, Znajewska Z, Dabrowska G. Use of rhizosphere microorganisms in the biodegradation of PLA and PET polymers in compost soil. Int Biodeterior Biodegrad. 2018;130:65–75.

[95] Wang F, Wang Q, Adams CA, Sun Y, Zhang S. Effects of microplastics on soil properties: Current knowledge and future perspectives. J Hazard Mater. 2022;424:127531. 34740160 10.1016/j.jhazmat.2021.127531

[96] Liu R, Liang J, Yang Y, Jiang H, Tian X. Effect of polylactic acid microplastics on soil properties, soil microbials and plant growth. Chemosphere. 2023;329:138504. 37011822 10.1016/j.chemosphere.2023.138504

[97] Chen L, Chang N, Qiu T, Wang N, Cui Q, Zhao S, Huang F, Chen H, Zeng Y, Dong F, Fang L. Meta-analysis of impacts of microplastics on plant heavy metal(loid) accumulation. Environ Pollut. 2024;348:123787. 38548159 10.1016/j.envpol.2024.123787

[98] Khaliq MA, Alsudays IM, Alhaithloul HAS, Rizwan M, Yong JWH, Rahman SU, Sagir M, Bashir S, Ali H, Hongchao Z. Biochar impacts on carbon dioxide, methane emission, and cadmium accumulation in rice from Cd-contaminated soils A meta-analysis. Ecotoxicol Environ Saf. 2024;274:116204. 38489905 10.1016/j.ecoenv.2024.116204

[99] Farhangi AS, Ghassemi-Golezani K. The modified biochars influence nutrient and osmotic statuses and hormonal signaling of mint plants under fluoride and cadmium toxicities. Front Plant Sci. 2022;13:1064409. 36578343 10.3389/fpls.2022.1064409 PMC9791105

[100] Wong JTF, Chow KL, Chen XW, Ng CWW, Wong MH. Effects of biochar on soil water retention curves of compacted clay during wetting and drying. Biochar. 2022;4:4–4.

