# Supplementary material for: Co-application of biochar and melatonin enhances rice resilience to combined lead and microplastics stress via modulating antioxidant defense, hormonal regulation, gene expression, and soil quality
Source: BMC Plant Biol. 2026 Apr 22;26:973. doi: 10.1186/s12870-026-08760-y (PMC13237945; doi:10.1186/s12870-026-08760-y)
Supplement: Supplementary file 1 — Supplementary Material 1. [file 12870_2026_8760_MOESM1_ESM.docx]

**Table S1:** List of primers used for gene expression analysis

| **Gene** | **Details of primers** |
| --- | --- |
| *OsAPX-F* | GGATGGGGCAAACCAGAAA |
| *OsAPX-R* | GGTCCTCATCTCGGCGTTC |
| *OsCAT-F* | CAACCACTACGACGGCTTCA |
| *OsCAT-R* | TCCTTGGCAATCACCACCTT |
| *OSPOX-F* | GTTGCCTGTTGATGCTCTGCT |
| *OsPOX-R* | CCGCCTGTGCTACGATGG |
| *OsSOD-F* | GCTGCCAGTTTGACTACAAATACC |
| *OsSOD-R* | AATGTGATCTCAGGCGACCC |
| *OsASMTI-F* | ATATTCCATGACGCGGGCTT |
| *OsASMTI-R* | TGGGTAAACCTCGATGACGG |
| *OsP5CS-F* | TGGAAGATTGGCTTTGGGCA |
| *OsP5CS-R* | CCCGGAACTTTGGGTTCTCA |
| *OsHMA9-F* | CAGTGAGCATCCTCTGGCAA |
| *OsHMA9-R* | GATGCTCCCGTCCTTTTGGA |
| *OsNRAMP5-F* | GAAGTGGCTTCGGAACCTGA |
| *OsNRAMP5-R* | GAAGCTCGTGCTCAGGAAGT |
| *Actin-F* | CATTGGTGCTGAGCGTTTCC |
| *Actin-R* | CCCGCAGCTTCCATTCCTAT |
